# An R-based reproducible and user-friendly preprocessing pipeline for CyTOF data

**DOI:** 10.12688/f1000research.26073.1

**Published:** 2020-10-22

**Authors:** Helena L. Crowell, Stéphane Chevrier, Andrea Jacobs, Sujana Sivapatham, Tumor Profiler Consortium, Bernd Bodenmiller, Mark D. Robinson

**Affiliations:** 1 Institute of Molecular Life Sciences, University of Zurich, Zurich, 8057, Switzerland; 2 SIB Swiss Institute of Bioinformatics, Zurich, 8057, Switzerland; 3 Department of Quantitative Biomedicine, University of Zurich, Zurich, 8057, Switzerland

**Keywords:** CyTOF, Preprocessing, Normalization, Debarcoding, Compensation, Gating, Batch correction, Reproducibility

## Abstract

Mass cytometry (CyTOF) has become a method of choice for in-depth characterization of tissue heterogeneity in health and disease, and is currently implemented in multiple clinical trials, where higher quality standards must be met. Currently, preprocessing of raw files is commonly performed in independent standalone tools, which makes it difficult to reproduce. Here, we present an R pipeline based on an updated version of CATALYST that covers all preprocessing steps required for downstream mass cytometry analysis in a fully reproducible way. This new version of CATALYST is based on Bioconductor’s SingleCellExperiment class and fully unit tested. The R-based pipeline includes file concatenation, bead-based normalization, single-cell deconvolution, spillover compensation and live cell gating after debris and doublet removal. Importantly, this pipeline also includes different quality checks to assess machine sensitivity and staining performance while allowing also for batch correction. This pipeline is based on open source R packages and can be easily be adapted to different study designs. It therefore has the potential to significantly facilitate the work of CyTOF users while increasing the quality and reproducibility of data generated with this technology.

## Introduction

Over the past decade, mass cytometry (CyTOF) has advanced our understanding of a wide range of cellular processes, particularly in the field of immunology and tumor biology ^1, 2^, by enabling the simultaneous measurement of 40+ parameters at the single cell level. Currently, mass cytometry is transitioning from an exploratory research approach toward a diagnostic tool used in clinical laboratories and this transition is associated with an increased need for standardization ^3^. Various studies have already suggested improvements on the experimental workflows to increase the robustness of mass cytometry data by working with frozen antibody cocktails or by including shared reference samples in each independent experiment to enable for batch correction ^4, 5^. Similarly, advanced downstream analyses benefit from the large number of analysis tools and algorithms implemented in R, which allow for fully reproducible analyses ^6^.

Between data generation and downstream data analysis, data preprocessing is a multi-step procedure required to convert raw FCS files into data objects that can be input to downstream statistical analysis and visualization ^7^. Upon data collection, the first step consists in concatenating files from sequential CyTOF runs and removing events of the acquisition with unstable signal, which are usually caused by uneven flow rate or introduction of air in the fluidic system. As a second step, CyTOF data need to be corrected for time dependent signal drift, which is mostly due to cone contamination, mass calibration drift or loss of detector sensitivity over time. This correction is performed by acquiring metal tagged polystyrene beads together with the cell suspension, where bead signals can be used as a reference to normalize the cell signals ^8^. Another potential artefact in CyTOF data is due to signal spillover between channels. Although lower than what is usually observed in fluorescent flow cytometry, spillover in mass cytometry can still account for up to 4% of the signal in some channels and needs to be corrected using signal compensation ^9^. Sample barcoding prior to staining is a common approach used in mass cytometry to combine multiple samples in a single experiment to minimize experimental variation due to staining and CyTOF acquisition. In this case, individual cells have to be assigned to their respective sample via a process called single cell debarcoding ^10^. In large studies where samples are collected over a long period of time by different users, on different machines or at different sites, an important step is to correct for batch effects, which can be achieved by including a shared control sample in each independent batch ^11, 12^. Finally, only live, intact single cells are relevant for the downstream analysis. Beads, doublets, debris and dead cells are excluded by gating on scatter plots ^7^.

Each step of the preprocessing pipeline requires expert decisions to determine the best parameters to achieve an optimal signal correction and cell selection. Moreover, all the chosen parameters should be recorded for reproducibility purposes. Despite these requirements, many current preprocessing pipelines still rely on switching between platforms that include, for example, MATLAB applications and (at least partially) closed source online platforms (e.g., Cytobank ^13^). This approach necessitates uploading the data to different platforms and carrying out certain steps in a purely manual fashion, which makes it time-consuming and difficult to reproduce. This is particularly limiting in a clinical setting, where reproducibility and large-scale data analysis are required. Thus, we propose a semi-automated R-based preprocessing pipeline for CyTOF data that is: i) fully reproducible; ii) includes quality checks and, iii) has limited need for supervision once the original setup has been made. This pipeline is developed around an updated version of CATALYST, an R package designed for preprocessing and differential analysis of mass cytometry data ^9, 14^. This new version of CATALYST is based on Bioconductor’s SingleCellExperiment class, the standard for high dimensional single cell data analysis. This pipeline can easily be adapted to each CyTOF user’s needs and will accelerate CyTOF data preprocessing while improving the quality of mass cytometry data generated.

### Data description

The data used in this pipeline were generated in the context of the Tumor Profiler project, a multi-center observational study investigating the relevance of different innovative technologies, including CyTOF, imaging mass cytometry, single-cell DNA and RNA sequencing, as well as *ex vivo* drug testing to improve the diagnostic of advanced cancer patients ^15^.

The samples of interest included blood samples and tumor biopsies collected at the University Hospital Zurich in spring 2020. These samples were assessed by mass cytometry in the context of a set of references including commercially available cell lines, PBMCs from healthy donors and PHA activated PBMCs. PBMCs from patients and healthy donors were collected based on a ficoll gradient ^16^, and tumor samples were dissociated as previously described ^17^. Once in single-cell suspension, all samples were stained for 5 min on ice with Cell-ID TM Cisplatin-194Pt (#201194, Fluidigm) to identify dead cells and subsequently fixed with PFA 1.6% (#15710, Electron Microscopy Sciences). Samples were stored as dry pellet at −80° C until CyTOF measurement.

The dataset used in this study was obtained from a single CyTOF run containing nine references, three blood samples and three tumor samples barcoded with a 20-well barcoding plate ^17^. Reference samples were selected to contain positive and negative populations for each marker included in the study’s antibody panel. This design was chosen to enable for quality control and batch correction across independent experiments based on quantile scaling as described in Schuyler *et al.* ^11^. After barcoding, pooled cells were stained at 4 degree for 1h with a 40-Ab panel designed to perform an in-depth characterization of the samples’ immune compartment. DNA intercalation was performed with a 1h room temperature incubation in Cell-ID Intercalator-Ir (#201192B, Fluidigm). Finally, the cell suspension was diluted 1:10 in Maxpar® Cell Acquisition Solution (#201240, Fluidigm) and 10% of EQ Four Element Calibration Beads (#201078, Fluidigm), and acquired on a Helios ^TM ^upgraded CyTOF 2 system at a flow rate of 150 events per second.

Throughout this workflow, we will make use of a set of metadata for standard preprocessing steps (normalization, debarcoding and compensation), as well as various quality controls. An overview of the metadata used is given in Table 1. All data used are provided as underlying data ^18^.

**Table 1.** Overview of metadata files used throughout this pipeline, including each file’s description, dimensionality (if appropriate), and purpose for preprocessing or quality control.

Description	Purpose
normalization_beads.fcs Beads identiﬁed using CATALYST during the normalization step of a previous CyTOF run.	Used as reference beads to correct for changes in signal sensitivity over time across multiple CyTOF runs.
ref_bead_counts.csv A table of mean dual counts for the six different bead channels (columns) obtained from 12 previous acquisitions (rows).	Used as a reference to assess the measurement sensitivity for the current run.
debarcoding_scheme.csv A binary barcoding scheme of 6- *choose*-3 = 20 barcodes with columns corresponding to barcode channel masses (101, 104, 105, 106, 108, 110) and rows corresponding to barcodes (7 empty, 9 references, 2 PBMC and 2 tumor samples)	Used for single-cell deconvolution of multiplexed samples.
spillover_matrix.csv A spillover matrix calculated with CATALYST from beads single-stained with each of the 40 antibodies included in the panel used in this study. The matrix contains, for each measurement channel (rows), the percentage of signal received by all other channels (columns).	Used for correction of spillover.
ref_cell_counts.csv A table of the number of cells measured in 7 previous acquisitions, each including 4 cell line, 3 PBMC and 2 tumor references samples (63 samples in total).	Used to asses reference sample cell yields in the current in comparison to previous runs.
sample_cell_counts.csv A table of the number of cells measured in 7 previous acquisitions, each including 2 PBMC and 2 tumor samples each (28 samples in total).	Used to asses sample cell yields in the current in comparison to previous runs.
ref_marker_levels.csv A table of the 98th expression percentiles for each target (columns) across 7 previous acquisitions (rows).	Used to assess the staining efﬁciency of the current run.

### Data organization

Most data used and returned throughout this workflow are kept in an object of Bioconductor’s SingleCellExperiment (SCE) class from the SingleCellExperiment package ^19^. This data structure can store all single-cell related data (measurement data and transformations thereof; cell, feature and experiment-wide metadata; dimensionality reductions), allowing for synchronized and thus less error-prone data manipulation.

The key component of SCEs are matrix-like assays, where rows are features (targets) and columns are observations (cells), that store the measurement data and any data derived thereof. Metadata associated with cells are stored under colData, feature metadata under rowData, and any experiment-wide metadata may be stored in the metadata slot. Lastly, the SCE can store an arbitrary number of dimensionality reductions under reducedDims. For a more detailed description of usage and structure of SCEs, we refer to the *SingleCellExperiment* package’s documentation.

## Results

The pipeline presented here describes all steps required to process raw mass cytometry data to a state where the user may proceed with downstream analyses (e.g., dimensionality reduction, differential analysis, trajectory inference). The process includes the concatenation of the individual acquisitions, the exclusion of part of the acquisition with unstable signal, the correction for time-dependent signal drift via bead normalization, the correction for signal spillover via compensation, the selection of cells of interest via automated gating, and the correction for batch effects. The workflow is exemplified on data obtained from three successive CyTOF acquisitions of 15 barcoded samples mixed with calibration beads.

We use CATALYST ^9^ to perform key preprocessing steps, including: concatenation, normalization, debarcoding and compensation; openCyto ^20^ and flowWorkspace ^21^ for gating; ggplot2 ^22^ and ggcyto ^23^ for visualization; flowCore ^24^ and dplyr ^25^ for data accession and manipulation; and mvtnorm to compute polygonal live gates. Thus, our workflow is limited to the following dependencies:



library(CATALYST)
library(dplyr)
library(flowCore)
library(flowWorkspace)
library(ggcyto)
library(ggplot2)
library(mvtnorm)
library(openCyto)


Besides standard preprocessing steps, we include quality control (QC) steps to assess CyTOF sensitivity, staining efficacy, and cell yield; these rely on results from previous runs ( *n >* 7) as a reference. For consistent visualization, we define a common plotting theme for boxplots that are used to compare the current to previous acquisitions:


qc_theme <- list(
  theme_bw(base_size = 8), theme(
    panel.grid.minor = element_blank(),
    panel.grid.major.x = element_blank(),
    plot.title = element_text(face = "bold"),
    axis.text = element_text(color = "black"),
    axis.text.x = element_text(angle = 45, hjust = 1)))


### Constructing a SingleCellExperiment

By default, *flowCore*’s read.FCS() function, which underlies read.flowSet() for reading in a set of FCS files, transforms channel intensities and removes events with extreme values. To omit this behavior, we recommend reading in files with arguments transformation = FALSE and truncate_max_range = FALSE; by default, files will be read in by CATALYST’s prepData() function with these settings.

As described above, the SCE class allows the keeping of multiple data transformations in a single object. Thus, when applying a transformation to arrive at expression-like data, we can store the transformed data in a separate assay without overwriting the raw ion count data. In this way, any data generated and used throughout preprocessing (e.g., normalized, compensated or batch-corrected counts and their arcsinh-transformed counterparts) can be in principal retained, and written to intermediate FCS files for backup or quality control outside of R. However, it is worth noting that this procedure could lead to a shortage of memory for large datasets, in which case overwriting the data at each step is advisable; if not specified otherwise, CATALYST overwrites by default.

A SCE can be constructed using CATALYST’s prepData() function, which accepts a path to a directory with one or many FCS files, a character vector of FCS filenames, a single or list of flowFrame(s), or a https://bioconductor.org/packages/3.11/bioc/html/flowCore.html ( *flowCore* package ^24^). By default ( transform = TRUE), an arcsinh-transformation with cofactor = 5 is applied to the input (count) data, and the resulting expression matrix is stored in the exprs assay slot of the output SCE:


# construct ’SingleCellExperiment’
fcs <- list.files("data", "acquisition", full.names = TRUE)
(sce <- prepData(fcs, transform = TRUE, cofactor = 5))



## class: SingleCellExperiment
## dim: 63 368152
## metadata(1): experiment_info
## assays(2): counts exprs
## rownames(63): 75As CD15 ... 208Pb CD45
## rowData names(4): channel_name marker_name marker_class use_channel
## colnames: NULL
## colData names(1): sample_id
## reducedDimNames(0):
## altExpNames(0):


Initially, our SCE has two assays containing dual ion counts (assay counts) and cofactor-5 arcsinh-transformed counts (assay exprs). The cofactor used for transformation is stored inside the object’s internal metadata ( int_metadata(sce)$cofactor), and the FCS file of origin for each cell under cell metadata column sample_id (accessible via colData(sce)$sample_id or, equivalently, sce$sample_id). In our dataset, FCS files correspond to acquisitions rather than biological samples. Thus, we rename the cell metadata variable sample_id to file_id to avoid ambiguity:


i <- match("sample_id", names(colData(sce)))
names(colData(sce))[i] <- "file_id"


The total number of cells across all acquisitions corresponds to the number of columns in the SCE ( ncol(sce)). We can summarize the number of cells in each file by tabulating the file_ids:


data.frame(
     file_id = levels(sce$file_id),
     n_cells = tabulate(sce$file_id))



##                   file_id n_cells
## 1 CyTOF_acquisition_1.FCS   48675
## 2 CyTOF_acquisition_2.FCS  125607
## 3 CyTOF_acquisition_3.FCS  193870


In both mass and flow cytometry, each feature has both a channel and target associated with it. As can be seen from printing the sce variable above, prepData() defaults to using targets as rownames (when available). We can retrieve each feature’s measurement channel using the channels() accessor, and use channel metals and masses to extract the indices of features that are relevant to different preprocessing steps. Namely, we assign channels measuring DNA to the variable dna, and channels for live gating (one DNA channel and cisplatin) to live:


# store character vector or channels names
chs <- channels(sce)
# store DNA & live channel indices
dna <- grep("^Ir", chs)
live <- grep("191|194", chs)


### Filtering for stable signal

**Figure 1.**
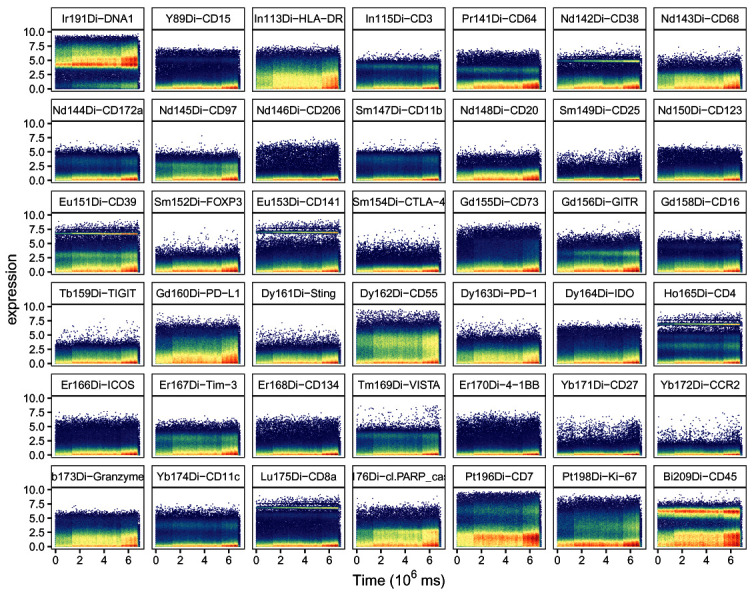
Scatter plots of DNA channel Ir191 and the 41 channels measuring antigens against time. Bins are colored by cell density; y-axis corresponds to cofactor-5 arcsinh-transformed dual counts.

High quality data generation requires a stable signal throughout the acquisition. Various issues can lead to signal change over time, including unstable flow rate, introduction of air or introduction of metal contamination in the system. These changes in signal intensity can vary in terms of duration and intensity, and can affect all or only a subsets of channels simultaneously. In order to detect regions of the acquisition affected by signal instability, we display the signal for selected channels as a function of time in a scatter plot ( Figure 1).


# plot channels of interest vs. time
coi <- chs[c(dna[1], which(rowData(sce)$use_channel))]
plotScatter(sce, chs = c("Time", coi), label = "both") +
     labs(y = "expression") +
     scale_x_continuous(
          expression("Time ("*10^6~"ms)"),
          labels = function(u) u/1e6) +
     theme_bw(base_size = 8) + theme(
          aspect.ratio = 2/3,
          panel.grid = element_blank(),
          axis.text = element_text(color = "black"),
          strip.background = element_rect(fill = NA))


In this particular experiment, we do not observe time-related signal instability. In case part of the acquisition should be excluded, this could be done by manually gating on the region with stable signal, and subsequent subsetting to only retain cells that fall within the gate’s boundaries (argument pop = "+"). Vice versa, it is possible to select a region with unstable signal, and remove it from the SCE object ( pop = "-"). For the sake of completeness, we include how a region of unstable signal could be excluded via manual gating:



# construct ’GatingSet’
ff <- sce2fcs(sce[dna, ], assay = "exprs")
gs <- GatingSet(flowSet(ff))

# apply rectangular gate to exclude unstable signal
min_t <- ...
max_t <- ...
gs_add_gating_method(
    gs, alias = "stable",
     pop = "-", parent = "root",
     dims = paste0("Time,", chs[dna[1]]),
     gating_method = "boundary",
     gating_args = sprintf("min=c(%s,0),max=c(%s,10)", min_t, max_t))

# plot scatter of DNA vs. Time
ggcyto(gs,
     aes_string("Time", chs[dna[1]])) +
     geom_hex(bins = 128) +
     geom_gate("stable") +
     facet_null() + theme_bw() +
     ggtitle(NULL) + theme(
         legend.position = "none",
         panel.grid = element_blank())

# subset selected events
sce <- sce[, gh_pop_get_indices(gs, "stable")]



### Normalization

In the case of mass cytometry, signal drift during acquisition due to a progressive loss of sensitivity must be accounted and normalized for. A widely established strategy is to mix samples with polystyrene beads embedded with metal lanthanides, allowing monitoring of instrument performance throughout data acquisition ^8^. These beads are in turn used to estimate and correct for the signal’s time drift. When independent experiments have to be analyzed in the same context, variation due to changes in instrument performance over time combined with intervals between scheduled maintenance have to be taken into account as well. In this case, the bead signal should be normalized to a set of reference beads from an earlier acquisition. This ensures that different experiments are normalized to the same level, independent of the CyTOF’s sensitivity.

A MATLAB tool to perform normalization outside of R was available until recently at nolanlab/beadnormalization; current R implementations are available through *CATALYST* and *premessa*. CATALYST provides an extension of bead-based normalization as described by Finck *et al.* ^8^, with automated identification of bead singlets (used for normalization), as well as of bead-bead and cell-bead doublets (to be removed), thus eliminating the need for manual gating. This is implemented as follows:

1. beads are initially identified as those events that have their highest signals in the bead channels
2. cell-bead doublets are removed by applying a separation cutoff on the distance between the lowest bead and highest non-bead channel signal
3. events passing all vertical gates defined by the lower bounds of bead signals are removed (these include bead-bead and bead-cell doublets)
4. bead-bead doublets are removed by applying a default *median* ± 5 *mad* rule to events identified in step 2; the remaining bead events are used for normalization

The above procedure is carried out by a single function, normCytof(), which takes as input a SCE and a set of arguments that control the normalization parameters and output format. Here, we specify beads = "dvs", corresponding to DVS Science beads (lanthanides Ce140, Eu151, Eu153, Ho165, Lu175). Secondly, we provide the path to a set of reference beads that are used to compute baseline intensities for normalization (argument norm_to). Lastly, we set overwrite = FALSE to retain both raw and normalized data, and remove_beads = TRUE to exclude bead and doublet events:


# specify path to reference beads
ref_beads <- file.path("data", "normalization_beads.fcs")
# apply bead-based normalization
system.time(res <- normCytof(sce,
     beads = "dvs", norm_to = ref_beads,
     remove_beads = TRUE, overwrite = FALSE))



##    user  system elapsed
##  18.567   1.155  19.930


When remove_beads = TRUE (the default), normCytof() will return a list of three SCEs containing filtered, bead and remove events, respectively, as well as two ggplot objects:


names(res)



## [1] "data"    "beads"   "removed" "scatter" "lines"


The first SCE ( res$data) contains the filtered data with additional assay slot "normed" housing normalized expressions. The remaining two SCEs are data subsets that contain any events identified as beads (slot beads) and all removed events (including beads, bead-bead and bead-cell doublets; slot removed), respectively; thus, the beads themselves are a subset of the removed events. Here, we compare the number and percentage of cells contained in each subset:


# view no. of remaining, bead & removed events
ns <- sapply(res[1:3], ncol)
ps <- sprintf("%1.2f", ns/ncol(sce)*100)
data.frame(t(cbind("# events" = ns, "% of total" = ps)))



##              data beads removed
## # events   337525 27303   30627
## % of total  91.68  7.42    8.32


**Figure 2.**
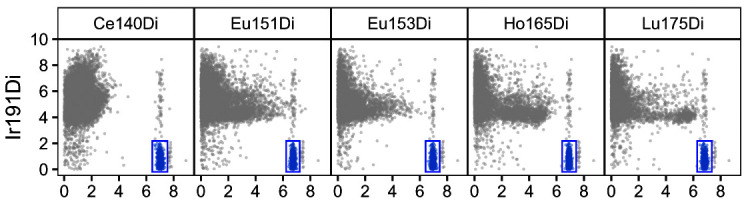
Scatter plots for bead channels vs. DNA. Events identified as beads are colored in blue; for each bead channel, expression ranges across all bead events are indicated as rectangular gates. Events were downsampled to at most 10,000 for visualization.

As a first quality control plot, res$scatter ( Figure 2) renders scatter plots of bead channels (x-axis) versus DNA (y-axis), where events identified as beads as well as their expression range are highlighted in color; bead events should have low DNA intensity (since they are not cells) and high intensities across all bead channels.

**Figure 3.**
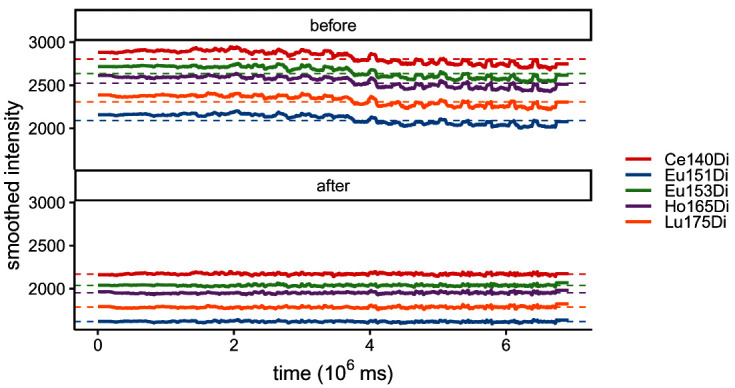
Running-median smoothed bead intensities vs. time before and after normalization; colored by bead channel.

Secondly, res$lines ( Figure 3) displays smoothed median bead intensities before and after normalization; these typically decrease with time prior to normalization, and should be approximately constant and centered around the baseline after normalization. In our dataset, normalization is performed based on previously acquired reference beads. Thus, baseline values correspond to the reference bead’s mean bead channel intensities. As shown in Figure 3, the bead channel levels are considerably lower after normalization, indicating higher sensitivity in the current run. Importantly, the slight decrease in signal over time is no longer present after normalization.

In order to assess the sensitivity of the CyTOF during acquisition and identify potential issues that would have remained undetected during the tuning of the instrument, we compute the mean bead channel counts across events identified as beads ( res$beads subset). A logical vector of which channels correspond to beads is stored under rowData column bead_ch, which we can use to subset the counts assay to include bead channels only.



# compute mean bead channel counts for current run
is_bead <- rowData(res$beads)$bead_ch   # get bead channels
bead_cs <- counts(res$beads)[is_bead, ] # subset counts
rownames(bead_cs) <- chs[is_bead]       # use channels as names
(bead_ms <- rowMeans(bead_cs))           # compute means

##  Ce140Di  Eu151Di  Eu153Di  Ho165Di  Lu175Di
## 2842.343 2111.072 2660.292 2537.831 2323.313


**Figure 4.**
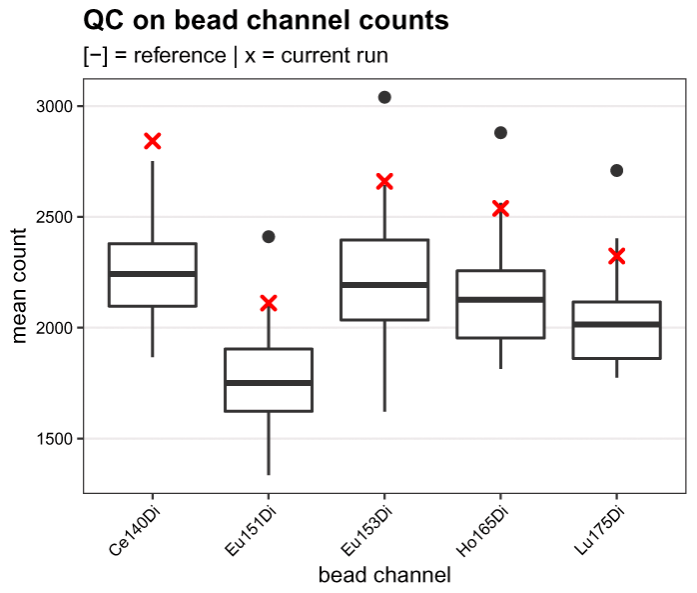
Bead channel count quality control. Boxplot comparing the mean dual ion counts (y-axis) across bead channels (x-axis) obtained for the current run (red crosses) to those from 12 previsouly acquired reference runs (boxes).

To assess the measurement sensitivity during the current run, we compare the mean bead channel counts computed above to those obtained from 12 previously acquired runs available in metadata table ref_bead_counts.csv. The resulting boxplot ( Figure 4) shows that the current run’s sensitivity is relatively high, but well in the range of previous runs.


# read in reference mean bead channel counts
ref <- read.csv(file.path("data", "ref_bead_counts.csv"))

# join into single tidy data.frame
df <- bind_rows(ref, bead_ms, .id = "group")
df <- melt(df, id.var = "group")

# boxplot of reference vs. current run’s mean bead channel counts
ggplot(df, aes(variable, value)) +
    geom_boxplot(data = df[df$group == 1, ]) +
    geom_point(data = df[df$group == 2, ],
        col = "red", pch = 4, stroke = 1) +
    labs(x = "bead channel", y = "mean count") +
    qc_theme + ggtitle(
        "QC on bead channel counts",
        "[-] = reference | x = current run")


After normalization, we overwrite the input dataset with the filtered subset that no longer includes bead events, or bead-bead and bead-cell doublets:


sce <- res$data


### Debarcoding

In mass cytometry, samples are often labeled with unique sample-specific barcodes and pooled together for processing and measurement, an approach termed *multiplexing* ^26^. The most widely used barcoding scheme is based on Zunder *et al.* ^10^, and relies on binary palladium-based mass-tag cell barcoding. Here, each sample *i* = 1, ..., *n* is either positive or negative for each of *m* palladium isotopes, resulting in an *m*-choose- *k* barcoding scheme, where *k* is the number of positive barcodes. For example, labeling of three out of six palladium isotopes will result in *n*-choose- *k* = 6-choose-3 = 20 unique barcodes. In order to recover the individual samples for further analysis, the pooled dataset is debarcoded (or *deconvoluted*) computationally.

The single cell debarcoding (SCD) algorithm first sorts each cell’s barcode intensities to assign preliminary barcode IDs such that a cell is assigned to the barcode population for which its barcode intensities are highest. Next, intensities within each barcode population are scaled using the 95th expression quantiles, and thereby brought to a comparable scale. Finally, events whose separation between highest negative and lowest positive barcode intensity is below a threshold value ( *separation cutoff*) are left unassigned.

In the initial SCD algorithm, sample yields are determined by a single global cutoff on the separation between positive and negative barcode populations. Naturally, this procedure is suboptimal when yields as a function of the applied cutoff do not decline simultaneously. To optimize cell yields in such cases, *CATALYST* provides an option to automatically estimate or specify *sample-specific* separation cutoffs.

The SCD algorithm is implemented in CATALYST as a three-step procedure: i) preliminary barcode assignment ( assignPrelim()); ii) automated estimation of sample-specific separation cutoffs ( estCutoffs()); and, iii) application of cutoffs to arrive at final barcode assignments ( applyCutoffs()).

### Preliminary barcode assigment

**Figure 5.**
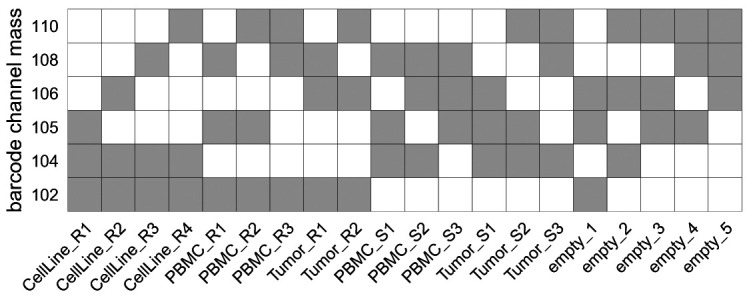
6-choose-3 palladium isotope debarcoding scheme. Rows correspond to palladium isotopes (barcode channels), columns to barcode identifiers (samples). Each sample is negative (white) or positive (grey) for 3 out of 6 barcode channels, resulting in 20 unique barcode combinations.



For our dataset, a 6-choose-3 = 20 barcoding scheme was used ( Figure 5). Five barcodes were unused ( empty_1-5), resulting in 15 samples (9 references, 6 samples of interest). We first read the corresponding debarcoding_scheme.csv into R:

# read in debarcoding scheme
fn <- file.path("data", "debarcoding_scheme.csv")
bc_key <- read.csv(fn, row.names = 1, check.names = FALSE)

# all barcodes are positive for exactly 3 barcoding channels
all(rowSums(bc_key) == 3)

## [1] TRUE


During this first debarcoding step, each event is preliminarily assigned to a barcode according to its top- *k* expressed barcode channels. Here, events whose expression is highest for a combination of barcode channels that does *not* appear in the debarcoding scheme ( bc_key) will be given barcode ID 0 (for “unassigned”). Thus, we can remove empty barcodes from the bc_key variable such that events assigned to these barcodes are left unassigned from the start. Alternatively, one could perform debarcoding using the non-subsetted key, and filter out empty barcodes downstream.


# remove empty barcodes from debarcoding scheme
is_empty <- grepl("empty", rownames(bc_key))
bc_key <- bc_key[!is_empty, ]
bc_ids <- rownames(bc_key)


For preliminary barcode assignment, we use CATLAYST’s assignPrelim() function, providing the input data ( sce) and debarcoding scheme ( bc_key). If not specified otherwise, assignPrelim() will default to using the exprs assay slot (argument assay). Because we ran normCytof() with overwrite = FALSE, this assay contains arcsinh-transformed *raw* counts; we set assay = "normexprs" in order to use the normalized values instead:


# do preliminary barcode assignments
system.time(sce <- assignPrelim(sce, bc_key, assay = "normexprs"))

##    user  system elapsed
##  15.150   0.394  15.685


In the returned SCE, feature metadata ( rowData) column is_bc indicates whether or not a channel corresponds to a barcode channel:


# view barcode channels
channels(sce)[rowData(sce)$is_bc]

##      MCB1      MCB2      MCB3      MCB4      MCB5      MCB6
## "Pd102Di" "Pd104Di" "Pd105Di" "Pd106Di" "Pd108Di" "Pd110Di"


For each event, barcode identifiers are stored in colData column bc_id. After this preliminary round of assignment, 57980/337525 events (17.18%) have been left unassigned:


# tabulate number of (un)assigned events
table(sce$bc_id == 0)

##
##  FALSE   TRUE
## 279545  57980


Furthermore, for each cell, the barcode channel expressions are scaled relative to the 95th expression percentiles of its respective barcode population. The scaled data is stored in assay slot scaled. Based on these scaled barcode channel intensities, a separation value is computed as the distance between highest negative and lowest positive barcode channel; separations are stored in colData column delta.

### Estimation of separation cutoffs

**Figure 6.**
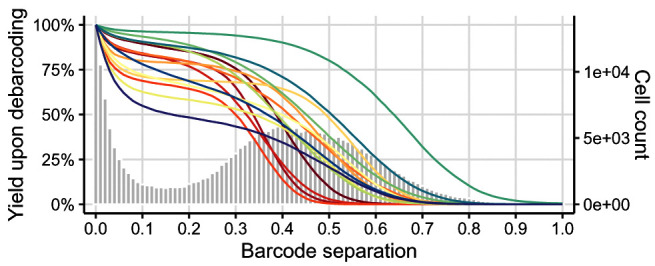
Yields plot for a 6-choose-3 debarcoding scheme. Shown is the distribution of barcode population separations (histogram) and cell yields by sample (lines) as a function of the applied separation cutoff. Left axis corresponds to cell yield in percent; right axis to the total number of cells.

To decide on separation cutoffs, we consider yields upon debarcoding as a function of the applied cutoff ( Figure 6). Commonly, this function will be characterized by an initial weak decline, where doublets are excluded, and subsequent rapid decline in yields to zero. In-between, low numbers of counts with intermediate barcode separation give rise to a plateau. Ideally, the applied separation cutoffs should provide a balance between high cell yield and low assignment uncertainty, marking the approximate midpoint of the yield function’s plateau region.


plotYields(sce, which = "0")


**Figure 7.**
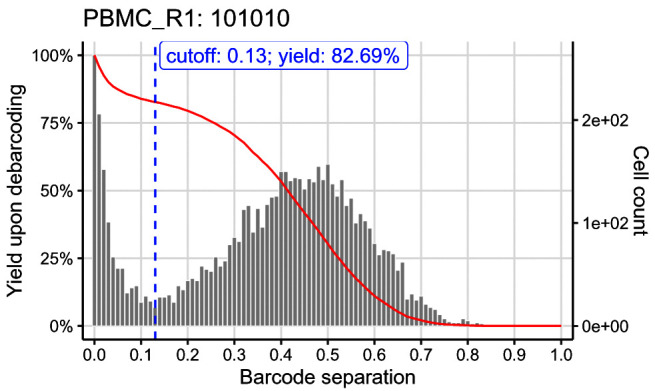
Yields plot for an exemplary sample, including the estimated separation cutoff. Shown is the distribution of barcode population separations (histogram) and cell yields (line) for the sample as a function of the applied separation cutoff. Left axis corresponds to cell yield in percent; right axis to the total number of cells.

Instead of a single global cutoff, we estimate a sample-specific cutoff to account for barcode population yields that decline in an asynchronous fashion. To this end, we fit both a linear and a three-parameter log-logistic model to each yield function. For the linear fit, we estimate the cutoff as the value for which yields have declined to 50%. For the log-logistic fit, we compute the cutoff as the value for which there is minimal yield decline by minimizing each yield function’s 1st derivative. For each barcode, the final cutoff estimate is computed as the mean of both estimates, weighted with the goodness (residual sum of squares) of each fit (see Methods for details). Thus, the choice of thresholds for the distance between negative and positive barcode populations is: i) automated and ii) independent for each barcode. Nevertheless, reviewing barcode-specific yield plots and, in rare cases, refining the estimated separation cutoffs is advisable (see Figure 7).

Cutoff estimation is performed by CATALYST’s estCutoffs() function, which takes as input a SCE as returned by assignPrelim(); that is, preliminary barcode assignments are required to estimate separation cutoffs. estCutoffs() will store sample-specific cutoff estimates under metadata slot sep_cutoffs, but will leave barcode assignments unchanged.



sce <- estCutoffs(sce)
metadata(sce)$sep_cutoffs

## CellLine_R1 CellLine_R2 CellLine_R3 CellLine_R4     PBMC_R1     PBMC_R2
##  0.13829757  0.13688667  0.09161242  0.12436944  0.13041729  0.18048328
##     PBMC_R3    Tumor_R1    Tumor_R2     PBMC_S1     PBMC_S2     PBMC_S3
##  0.26517571  0.21012223  0.20543857  0.10440146  0.12902187  0.24858758
##    Tumor_S1    Tumor_S2    Tumor_S3
##  0.18444497  0.14691641  0.20818002



We can visually inspect the estimated cutoffs using plotYields() with argument which specifying the barcode ID of interest ( Figure 7). In our example, the cutoff estimate nicely marks the midpoint of the yield function’s plateau or, equivalently, the valley between peaks of cell yields. To decrease the stringency of the applied cutoff, and thus increase the resulting cell yield, we could set the sample’s cutoff to e.g. 0.1. Vice versa, a more stringent cutoff of e.g. 0.2 would decrease the cell yield but yield a purer population.

As an alternative to inspecting the cutoff estimate for each sample in R, we could specify which = bc_ids to obtain a list of yield plots for all barcodes; the generated set of plots may be written to a single PDF file via providing plotYields() with an out_path to allow for easy reviewing of the separating cutoffs currently stored within the object.


plotYields(sce, which = "PBMC_R1")


Besides a cutoff on the separation between positive and negative barcode populations, to trim outliers, the SCD algorithms applies an additional cutoff on the *Mahalanobis distance* (argument mhl_cutoff), a metric that quantifies the distance of a given event to the expression distribution of the barcode population it has been assigned to.

In Figure 6, we can observe that population yields decline synchronously with increasing separation cutoffs, and that we might consider applying a global separation cutoff, e.g., at ∼ 0.15. For this data, yields are in fact similar, independent of whether we apply sample-specific cutoffs or a single global one. Nevertheless, applying sample-specific cutoffs is recommended in order to maximize cell yields while minimizing uncertainty in barcode assignments.


# store preliminary barcode IDs
bc_ids0 <- sce$bc_id

# apply global & sample-specific separation cutoff(s)
sce_glob <- applyCutoffs(sce, sep_cutoffs = 0.15, mhl_cutoff = 30)
sce_spec <- applyCutoffs(sce, mhl_cutoff = 30)

# compare cell yields for both cutoff strategies
c(global = mean(sce_glob$bc_id == 0),
  specific = mean(sce_spec$bc_id == 0))

##    global  specific
## 0.3573839 0.3584949


After debarcoding, we compare the number of events assigned to each barcode population before and after applying separation cutoffs, and filter out events that have been left unassigned (barcode ID 0). As shown in Figure 8, after applying the separation cutoffs, the number of unassigned cells (0) increases, while the number of cells assigned to each barcoding well decreases. We also observe a higher decrease in assigned cells for tumor samples, which underwent a dissociation process and contain more debris. Conversely, highly viable cell lines and PBMCs have a higher recovery yield.


# proceed with sample-specific filtering
sce <- sce_spec

# compute number of events per population
# before vs. after applying separation cutoffs
barplot(rbind(table(bc_ids0), table(sce$bc_id)),
     beside = TRUE, ylab = "cell count",
     las = 2, cex.axis = 0.5, cex.names = 0.5)
legend("topright", fill = c("black", "grey"),
     legend = c("before filtering", "after filtering"))



# remove unassigned events
sce <- sce[, sce$bc_id != 0]


**Figure 8.**
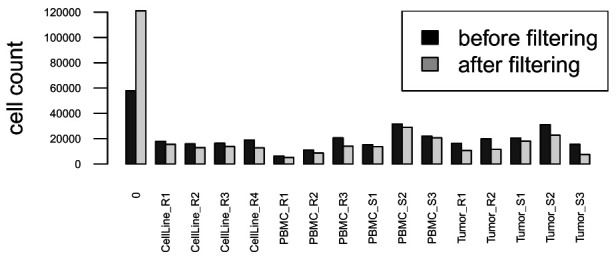
Barplot of cell counts before (black) and after (grey) apply separation cutoffs.

### Compensation

Mass cytometry utilizes heavy metals (usually from the lanthanide series) as reporters to label antibodies. As a result, channel crosstalk originating from spectral overlap and autofluorescence is significantly less pronounced in mass cytometry compared to flow cytometry. Yet, spillover due to abundance sensitivity, isotopic impurities, and oxide formation still exists, giving rise to artefactual signal that can impede data interpretability.

A combined experimental-computational pipeline to correct for spillover in mass cytometry data has been proposed by Chevrier *et al.* ^9^ and is implemented in the *CATALYST* package. In brief, compensation is achieved via the following three-step approach outlined here (see for details).

1. Identification of single positive populations via deconvolution of single-stained beads ( assignPrelim(), estCutoffs(), applyCutoffs()).
2. Estimation of a spillover matrix (SM) from the populations identified ( computeSpillmat()).
3. Compensation via multiplication of measurement intensities by the SM’s inverse, the compensation matrix ( compCytof()).

We will apply a pre-acquired spillover matrix (metadata file spillover_matrix.csv). Thus, we enter at step 3, which involves only compensating the input dataset using CATALYST’s compCytof() function. By default, compCytof() will reuse the cofactor stored in int_metadata(sce)$cofactor for computing arcsinh-transformed data from the compensated counts, thus applying the same transformation as during data preparation and normalization:



# read in pre-computed spillover matrix
sm <- file.path("data", "spillover_matrix.csv")
sm <- read.csv(sm, row.names = 1)
# apply NNLS compensation
system.time(
     sce <- compCytof(sce, sm, method = "nnls",
          assay = "normcounts", overwrite = FALSE))

##    user  system elapsed
##  67.525   7.318  75.337


**Figure 9.**
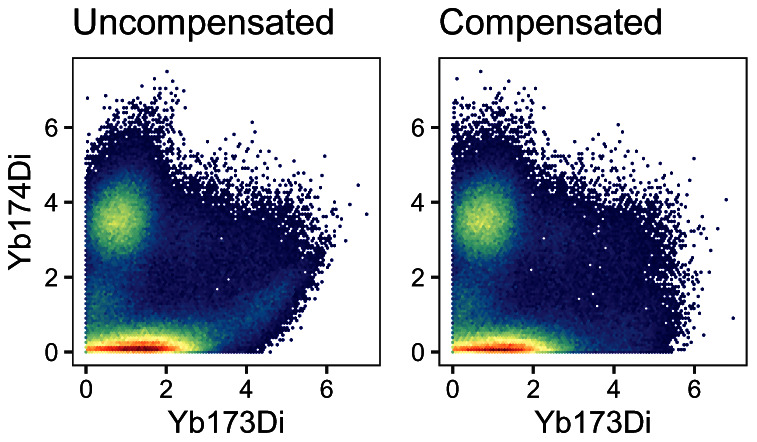
Scatter plots for two exemplary channels before (left) and after correction for spillover (right).

To visually inspect how compensation affects signal intensities, we can generate scatter plots pre- and post-compensation; an exemplary pair of channels is shown in Figure 9. In such a plot, we can observe a slight positive association between the signals of spill-affected channels, which should be removed upon compensation.


i <- grep("173|174", chs, value = TRUE)
p1 <- plotScatter(sce,
  chs = i,
  label = "channel",
  assay = "normexprs") +
  ggtitle("Uncompensated")
p2 <- plotScatter(sce,
  chs = i,
  label = "channel",
  assay = "compexprs") +
  ggtitle("Compensated") +
  ylab(NULL)
wrap_plots(p1, p2)


### Gating

Many events acquired in mass cytometry may in fact be debris, doublets or dead cells, and should be filtered out through a gating step. Here, we suggest a strategy that first applies an elliptical gate on cell events, defined as double positive for the DNA channels Ir191/Ir193. This allows the exclusion of debris and doublets. As a second step, we discard cells that are positive for the dead cell marker Pt194.

These two steps are performed using the *openCyto* R package ^20^, and the resulting gates are visualized on scatter plots of the channels subjected to gating using *ggcyto* ^23^. For consistent visualization, we again define a common plotting theme for scatter plots of channels chs that include the gating boundaries for the specified gate_id:



.scatter <- function(gs, chs, gate_id = NULL,
    subset = ifelse(is.null(gate_id), "root", "_parent_")) {
    p <- ggcyto(gs, max_nrow_to_plot = 1e5,
        aes_string(chs[1], chs[2]), subset) +
        geom_hex(bins = 100) + facet_wrap(~ name, ncol = 5) +
        (if (is.null(gate_id)) list() else geom_gate(gate_id)) +
        ggtitle(NULL) + theme_bw(base_size = 8) + theme(
             aspect.ratio = 1,
             legend.position = "none",
             panel.grid.minor = element_blank(),
             strip.background = element_rect(fill = NA),
             axis.text = element_text(color = "black"),
             axis.text.x = element_text(angle = 45, hjust = 1))
    suppressMessages(p + coord_equal(expand = FALSE,
        xlim = c(-1, 11), ylim = c(-1, 11)))
}


### Gating on cells

In order to apply sample-specific gates, we first convert the SCE into a flowSet with a separate frame for each sample (argument split_by = "bc_id"). As gating should be performed on expression-like data (not ion counts), we further specify assay = "exprs" to retain the arcsinh-transformed assay slot. Thirdly, since conversion from SCE to flowCore data structures requires matrix transposition (rows correspond to targets in the SCE, but to events in flowFrame/Sets), we retain only those channels that are relevant when gating of (live) cells: DNA and dead channels, whose indices are stored in variables dna and live.


# subset DNA & live channels
sub <- sce[union(dna, live), ]

# add metadata variable ’i’ to track cell indices
colData(sub) <- DataFrame(
     bc_id = sub$bc_id,
     i = seq_len(ncol(sce)))

# split SCE by sample
fs <- sce2fcs(sub,
     assay = "compexprs",
     split_by = "bc_id",
     keep_cd = TRUE)

# construct ’GatingSet’
gs <- GatingSet(fs)


We apply an elliptical gate ( gating_method = "flowClust.2d") to exclude the two lowest density percentiles ( quantile = 0.98). Because the input gating set contains a separate frame for each barcode, the gate will be computed separately for each sample.


# apply elliptical gate on DNA channels
gs_add_gating_method(gs,
     alias = "cells",
     pop = "+", parent = "root",
     dims = paste(chs[dna], collapse = ","),
     gating_method = "flowClust.2d",
     gating_args = "K=1,quantile=0.98,target=c(5,5)")



We use *ggcyto* to produce scatter plots of the DNA channels, with geom_gate("cells") to visualize the gates computed above ( Figure 10):



# plot scatter of DNA channels split by sample
.scatter(gs, chs[dna], "cells")


**Figure 10.**
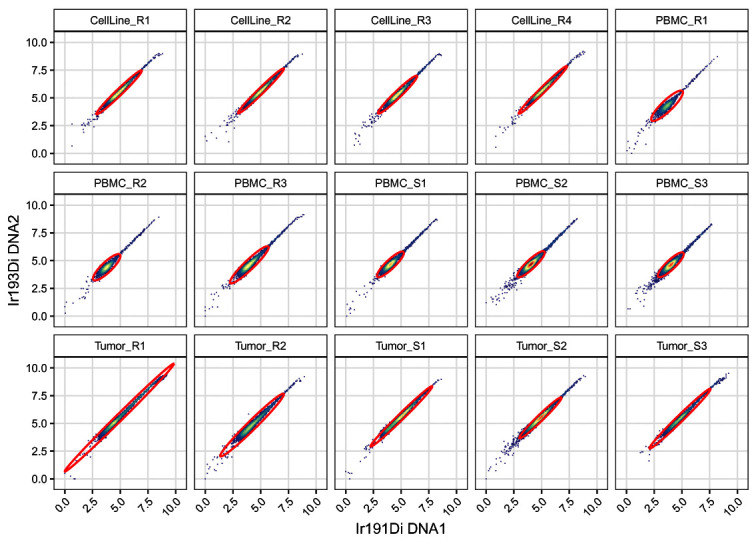
Scatter plots of DNA channels, split by sample and including elliptical cell gates.

### Gating on live cells

The wrapper function .live_gate() defines a polygonal gate comprised of a line and a bivariate standard normal density Z, such that cells pass gating when i) their expression is within the qth quantile of Z; and, ii) their expression falls below a line parameterized by intercept i and slope s. In this way, the gate is centered around the expression peak, while excluding cells whose dead channel intensities increases with DNA content.


# define live cell gate plug-in
# x = expression matrix, q = quantile, i = intercept, s = slope
.live_gate <- function(x, q = 0.99, i = 1, s = 0.5) {
    # specifying gating function
    .gating_fun <- function(fr, pp_res, channels = NA, id = "", ...) {
         # subset channels of interest
         x <- exprs(fr[, channels])
         # scale data for comparison w/ ’qnorm()’
         x0 <- scale(x)
         # set boundary level as q-th quantile of standard normal
         z <- qnorm(q)
         # find p(x) for that level
         pd <- dmvnorm(c(z, z))[1]
         px <- dmvnorm(x0)
         # find points above boundary level
         keep1 <- px > pd
         # find points below line y = a + b * x
         keep2 <- (i + s * x0[, 1]) > x0[, 2]
         # intersection of points below line & above threshold level
         pts <- x[keep1 & keep2, ]
         # get boundary points (convex hull)
         pts <- pts[chull(pts), ]
         # return gate
         polygonGate(.gate = pts, filterId = id)
    }
    # register gate
    suppressMessages(
      foo <- register_plugins(
        fun = .gating_fun,
        methodName = "liveGate",
        dep = "mvtnorm",
        "gating"))
}



**Figure 11.**
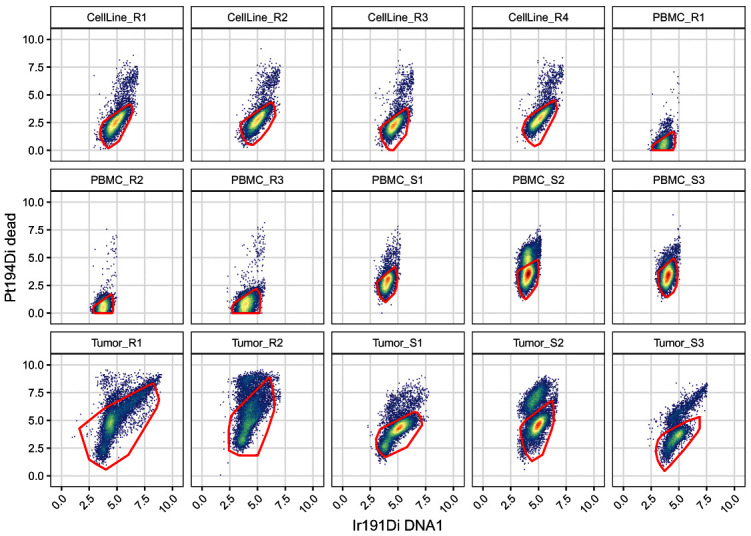
Scatter plots of DNA and dead cell channels, split by sample and including the dead cell polygon gates.

In contrast to the cell gates above, we apply live gates with sample-specific gating parameters. To this end, we specify a list l containing quantiles q, intercepts i and slopes s for each sample. These parameters are updated iteratively to remove dead cells while retaining cell yields as high as possible. After manual adjustments, we arrive at the following sample-specific gating parameters ( Figure 11).



# set default parameters for all samples
l <- lapply(c(q = 0.99, i = 0.9, s = 0.4), function(u)
    setNames(rep(u, length(gs)), sampleNames(gs)))

# adjust parameters for specific samples
l$i[["PBMC_R2"]] <- 1.2
l$i[["PBMC_R3"]] <- 1.2
l$i[["PBMC_S1"]] <- 1.2
l$s[["PBMC_S2"]] <- 0.2
l$i[["PBMC_S2"]] <- 0.6
l$i[["PBMC_S3"]] <- 1.8
l$s[["Tumor_S2"]] <- 0.3
l$i[["Tumor_S2"]] <- 0.6
l$s[["Tumor_S3"]] <- 0.3
l$i[["Tumor_S3"]] <- 0.4

for (i in sampleNames(gs)) {
    # register & apply live gate with sample-specific parameters
    .live_gate(x, q = l$q[i], i = l$i[i], s = l$s[i])
    gs_add_gating_method(gs[i],
         alias = "live",
         pop = "+",
         parent = "cells",
         dims = paste(chs[live], collapse = ","),
         gating_method = "liveGate")
}
.scatter(gs, chs[live], "live")


**Figure 12.**
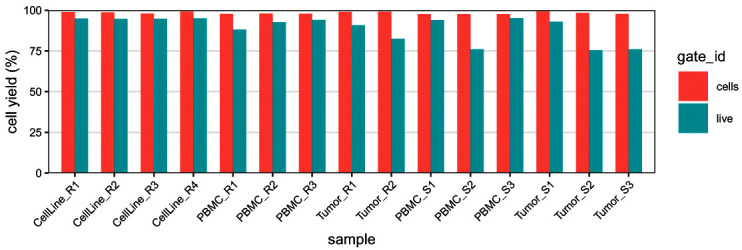
Barplot of cell and live gating yields. For each barcode ID (x-axis), frequencies are relative to the total number of cells in the population before gating; bars are colored by gate ID.

We display the yield of "cell" and "live" gates on each samples to quickly assess the cell losses occurring at the two gating steps ( Figure 12). As expected the "cell" gate leads to a systematic loss of around 1% of cells across all the samples. The "live" gate leads to a stronger reduction of cell yield in the tumor samples, consistent with the fact that those samples, which underwent enzymatic dissociation, contain more dead cells.


# extract gating frequencies
df <- gs_pop_get_stats(gs,
    type = "percent",
    nodes = c("cells", "live"))
df <- rename(df, gate_id = "pop")

# barplot of cell yields after cell/live gating
ggplot(df, aes(sample, percent, fill = gate_id)) +
     geom_bar(width = 2/3, stat = "identity", position = "dodge") +
     scale_x_discrete(limits = bc_ids, expand = c(0, 2/3)) +
     scale_y_continuous(labels = seq(0, 100, 25),
          limits = c(0, 1), expand = c(0, 0)) +
     labs(y = "cell yield (%)") + qc_theme



We extract a logical vector indicating whether a given event is included in or excluded by the "live" gate applied above by applying gh_pop_get_indices to each sample in gs. Secondly, we extract the cell indices from gs and subset the SCE to keep only cells that passed the "live" gate.



fs <- gs_pop_get_data(gs, "live") # get data from ’GatingSet’
es <- lapply(fs, exprs)            # get expression matrices
es <- do.call("rbind", es)         # join into single data.frame
sce <- sce[, es[, "i"]]            # subset retained cells


**Figure 13.**
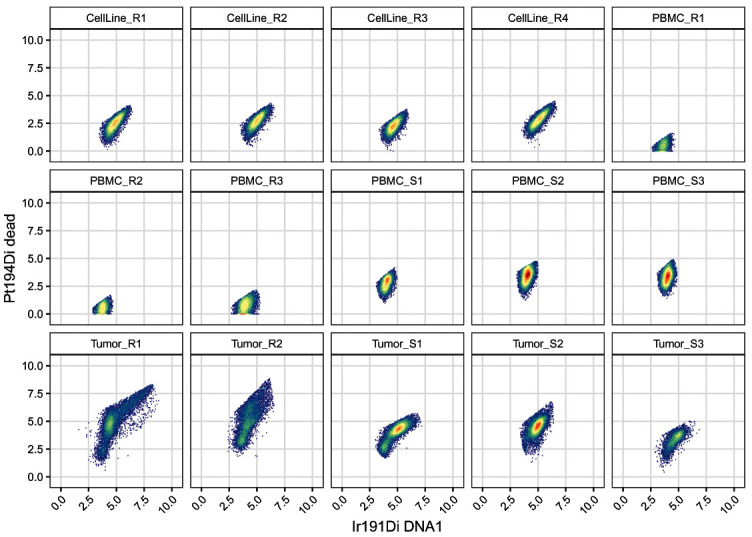
Scatter plots of dead cell channels against DNA, including the subset of cells remaining after live cell gating.

Finally, we can again visualize scatter plots of dead channels against DNA as a quality control for the retained subset of cells ( Figure 13).

### Quality control

Having completed the standard preprocessing steps, we proceed to investigate how the current run compares to prior acquisitions in terms of the number of cells in each reference and sample, and the expression levels of each target. Large parts of the metadata generated by now may no longer be needed, and unnecessarily increases output file sizes for large-scale datasets. Therefore, we will retain only two key cell metadata variables: file_ids containing the FCS filename each cell originates from, and bc_ids containing the barcode population assignments. We secondly rename these variable to make the following quality control steps more intuitive.


# drop all cell metadata except file of origin & barcode IDs
colData(sce) <- colData(sce)[c("file_id", "bc_id")]

# rename cell metadata variable
i <- match("bc_id", names(colData(sce)))
names(colData(sce))[i] <- "sample"


In the debarcoding scheme used for deconvolution of the multiplexed samples (Section Debarcoding), barcode identifiers were chosen to contain all information relevant for each sample. This setup allows us to extract sample metadata directly from the bc_ids. Alternatively, and especially for more complex experimental designs, this information could be stored in a separate metadata table. Such a table could then be used to match the bc_ids with the listed samples, and add arbitrary metadata information (e.g., batch, patient ID, treatment).

In our example, barcode identifiers include each sample’s type ( CellLine, PBMC or Tumor), group ( R for reference or S for sample of interest), and replicate number; and follow a consistent naming scheme: . We can easily extract these components and store them in the SCE’s cell metadata ( colData):


sce$type <- gsub("_.*", "", sce$sample)
sce$group <- gsub("[^R|S]", "", sce$sample)
head(colData(sce))



## DataFrame with 6 rows and 4 columns
##                   file_id      sample        type       group
##                  <factor> <character> <character> <character>
## 1 CyTOF_acquisition_1.FCS CellLine_R1    CellLine           R
## 2 CyTOF_acquisition_1.FCS CellLine_R1    CellLine           R
## 3 CyTOF_acquisition_1.FCS CellLine_R1    CellLine           R
## 4 CyTOF_acquisition_1.FCS CellLine_R1    CellLine           R
## 5 CyTOF_acquisition_1.FCS CellLine_R1    CellLine           R
## 6 CyTOF_acquisition_1.FCS CellLine_R1    CellLine           R



### Quality control (QC) on reference cell counts

**Figure 14.**
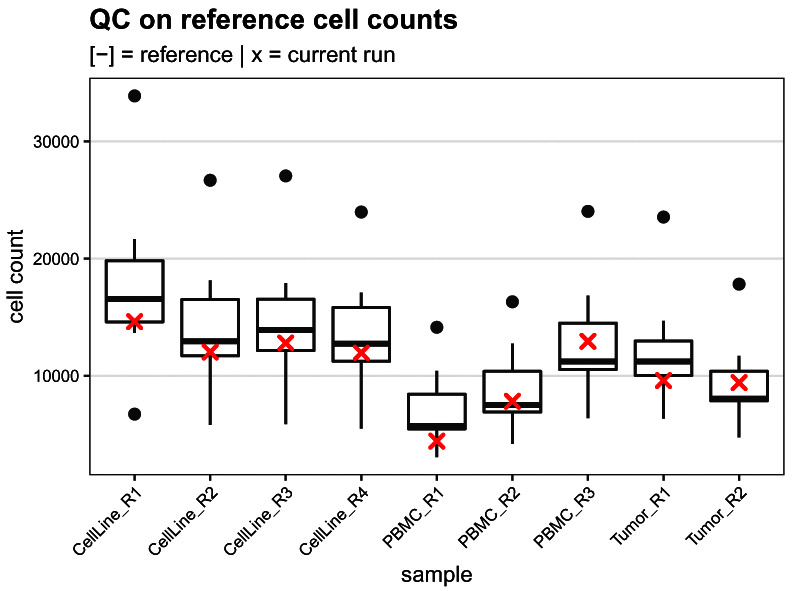
Reference cell count quality control. Boxplot comparing the reference cell counts obtained for the current run (red crosses) to those from 7 previsouly acquired runs.

As a first quality control, we compare the cell counts of each reference sample ( R) to those obtained from 7 previous runs ( Figure 14). Since the references are obtained from pre-barcoded aliquots of cells, the number of reference cells acquired gives direct information regarding the cell yield throughout the whole experiment: From cell barcoding to acquisition on the CyTOF. As shown in Figure 10, the current run has a lower yield compared to average run. This observation is consistent across all references measured.


# boxplot of current vs. reference cell counts
ref <- read.csv(file.path("data", "ref_cell_counts.csv"))
run <- table(sce$sample[sce$group == "R"])

# join into single tidy data.frame
df <- bind_rows(ref, run, .id = "group")
df <- melt(df, id.var = "group")

ggplot(df, aes(variable, value)) +
     geom_boxplot(data = df[df$group == 1, ]) +
     geom_point(data = df[df$group == 2, ],
         col = "red", pch = 4, stroke = 1) +
     labs(x = "sample", y = "cell count") +
     qc_theme + ggtitle(
         "QC on reference cell counts",
         "[-] = reference | x = current run")


### QC on sample cell counts

**Figure 15.**
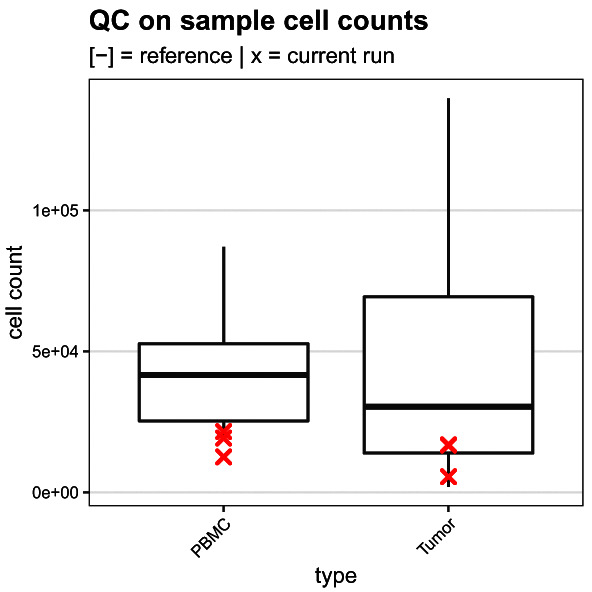
Sample cell count quality control. Boxplot comparing the sample cell counts obtained for the current run (red crosses) to those from 7 previsouly acquired runs.

Secondly, we compare the cell counts for the 4 samples of interest (2 PBMC, 2 tumor samples) to the number of cells recorded for 14 tumor and PBMC samples each (28 reference samples in total) acquired in previous runs ( Figure 15). This step provides a first quality assessment of the samples of interest. Here, samples with too few cells will be less reliable, and potentially less representative of the original tissue, making conclusions from downstream analyses more difficult to draw.


ref <- read.csv(file.path("data", "sample_cell_counts.csv"))
run <- table(sce$sample[sce$group == "S"], dnn = "sample")
run <- as.data.frame(run, responseName = "count")
run$type <- sce$type[match(run$sample, sce$sample)]
df <- bind_rows(ref, run, .id = "group")

ggplot(df, aes(type, count)) +
     geom_boxplot(data = df[df$group == 1, ]) +
     geom_point(data = df[df$group == 2, ],
         col = "red", pch = 4, stroke = 1) +
     labs(x = "type", y = "cell count") +
     qc_theme + ggtitle(
         "QC on sample cell counts",
        "[-] = reference | x = current run")


### QC on mean marker intensities

As the third and final quality control, we compare the 98th expression quantiles across all targets of interest over the pooled references to those obtained from 8 previously acquired runs available in metadata table ref_marker_levels.csv ( Figure 15). We chose the 98th percentile to account for the fact that some populations are rare, and we are particularly interested in assessing signal stability for positive cells rather than the median of the population. Since the pooled references are identical from one run to another, this gives a direct indication of the current run’s staining efficacy and enables early identification of antibody degradation over time.


# read in reference data
ref <- file.path("data", "ref_marker_levels.csv")
ref <- read.csv(ref, check.names = FALSE)

# compute 98th expression quantiles
# for reference samples in current run
es <- assay(sce, "compexprs")
es <- es[names(ref), sce$group == "R"]
run <- rowQuantiles(es, probs = 0.98)

# join into single tidy data.frame
df <- bind_rows(ref, run, .id = "group")
df <- melt(df, id.var = "group")

ggplot(df, aes(variable, value)) +
     geom_boxplot(data = df[df$group == 1, ]) +
     geom_point(data = df[df$group == 2, ],
         col = "red", stroke = 0.5) +
     labs(x = "target", y = "98th expression quantile") +
     qc_theme + ggtitle(
         "QC on marker levels",
         "[-] = referece | o = current run")


**Figure 16.**
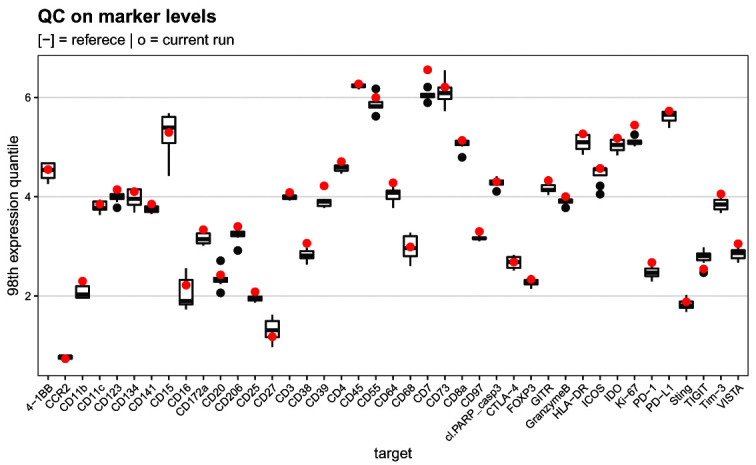
Mean marker expression quality control. Boxplot comparing the mean marker expression obtained for the current run (shown in red) to those from 8 previsouly acquired runs.

### Batch alignment

Each CyTOF run contains the same set of references. Similar to the approach used by Schuyler *et al.* ^11^, we use these references as anchors to calculate a channel-specific correction factor by dividing the 98th percentile measured in the current run by the average 98th percentile obtained across the first seven runs of the project. The signal observed in each channel for the samples of interest is then divided by these correction factors derived from the reference samples ( Figure 16).


# compute 98th count quantiles via back-transformation
# (using same cofactor as always) & average across replicates
cf <- int_metadata(sce)$cofactor
ref <- data.frame(
  target = colnames(ref),
  mean_count = colMeans(sinh(ref)*cf))

# initialize correction factor of 1 for all channels
cfs <- setNames(rep(1, nrow(sce)), rownames(sce))

# compute batch correction factors for relevant channels
cs <- assay(sce, "compcounts")
csR <- cs[ref$target, sce$group == "R"]
run <- rowQuantiles(csR, probs = 0.98)
cfs[ref$target] <- run / ref$mean_count

# apply marker-specific batch correction (bc)
cs <- sweep(cs, 1, cfs, "/")
assay(sce, "bccounts") <- cs

# apply arcsinh-transformation
assay(sce, "bcexprs") <- asinh(cs/cf)


## Discussion

In this workflow, we have presented a pipeline for reproducible and highly-automated preprocessing of CyTOF data, based on an updated version of CATALYST. Our pipeline covers four standard steps: Normalization for signal time-drift using bead standards (Section Normalization), single-cell deconvolution of multiplexed samples (Section Debarcoding), correction for spillover via compensation (Section Compensation), and gating for live cells (Section Gating). Moreover, we have included various quality control steps that compare the current acquisition to a set of reference data (Section Quality control). These steps ensure that measurement sensitivity, gating cell yields, sample cell counts, and expression levels lie within the expected range.

A key advantage of both using and developing Bioconductor packages is that they utilize common data structures, thereby greatly facilitating interaction between them. For example, many of the data structures used in scRNA-seq data analysis have only become established relatively recently. Meanwhile, the cytometry community has been relying on the FCS file format for data storage, and *flowCore*’s flowFrame/flowSet as well as *flowWorkspace*’s GatingSet classes for computational analyses. While there exists a lot of infrastructure around these data structures, they impede method development for newly emerging standards, and act as a barrier for interpolation of analyses across currently developed packages. This is particularly visible in the context of other fast growing single-cell data types such as scRNA-seq data analysis, where most current methods are being developed around Bioconductor’s SingleCellExperiment class. To name just two examples, an extensive collective of visualization tools for SCEs is available through *scater* ^27^, including a variety of dimensionality reduction methods; and methods for differential abundance (DA) analysis (to detect subpopulations that are differently abundant between conditions) and differential state (DS) analysis (to test for subpopulation-specific expression changes across conditions) are implemented in *diffcyt* ^28^.

The SCE class allows storing multiple assays that can, for example, contain raw counts, expression-like data obtain upon arcsinh-transformation, as well as any intermediate data matrices obtained after normalization, compensation and batch correction. Moreover, any event (cell) and feature (marker) metadata generated in the process can be added to the object’s colData/rowData, alongside an arbitrary number of dimensionality reductions. Thus, SCEs present an overall more compact and less error-prone data structure for both preprocessing and downstream analysis when compared to storing the various data matrices or metadata in separate variables, which would have to be combined for certain computations, separately subsetted and saved to independent outputs.

There is an obvious benefit for the mass cytometry community to take advantage of these new infrastructure developments. However, it is equally important to maintain backward compatibility with well-established standards in the field. For example, it can be desirable to write out intermediate outputs (FCS files) after each proprocessing step, or make use of available tools that build around flowCore’s flowFrame and flowSet classes, or other classes derived thereof (e.g., *flowWorkspace*’s GatingSet). Thus, while CATALYST’s transition to a more recent and an arguably advantageous data structure is motivated by the ability to leverage many existing and newly-developed tools, a complete dismissal of the large infrastructure that is available in the realm of cytometry data analysis is impossible at this time. To facilitate conversion between SCEs and conventional cytometry data structures, CATALYST provides the sce2fcs() function, which allows the user to specify which assay data to retain, whether to drop or keep available cell metadata and dimensionality reductions, and (optionally) to split the input dataset by a non-numeric variable (to, e.g., export each sample to a separate FCS file).

Although the current version of this pipeline constitutes a comprehensive approach to generate high-quality data for downstream analysis, further developments could be added in the future. In particular, it could be useful to implement an automated way of identifying and removing part of the data with unstable signal, similar to the approach proposed by *flowClean* ^29^, an R package designed to exclude fluorescent anomalies in flow cytometry data. Given that selection of anomalies in the dataset by the user is subjective, or that they may be altogether undetectable by eye, the advantage of such an approach would be to further standardize the process while decreasing manual work.

Recently, batch normalization has become of increased importance in order to enable integration of datasets acquired at different times, by different users and on different instruments. Here, we use scaling normalization where references are used as anchors to correct all samples included in the analysis in a channel specific way, similar to the strategy proposed by Schuyler *et al.* ^11^. While this approach requires a well-defined experimental procedure where references with positive and negative subsets for each marker have to be included in each acquisition, it does not make any assumptions on sample compositions. Thus, since the dataset used in this pipeline was acquired on the same instrument and stained with the same frozen antibody panel as previous acquisitions, scaling by expression quantiles provides an efficient way to correct for batch effects. To increase the flexibility of batch correction in cases where the experimental variation is higher, CATALYST could integrate different methods that have the potential to increase batch correction efficiency. For example, *CytoNorm* ^12^ computes quantiles for every metacluster and for every marker after aggregation of control samples from each batch. Such an approach could be more appropriate in cases where the references’ expression distributions are less aligned. An alternative method, *CytofRUV* ^30^, exploits the concept of pseudo-replicates to remove unwanted variation (RUV) between proteins and cells. Here, cells are grouped into subpopulations using *FlowSOM* ^31^ clustering. Groups of cells present across all batches are considered to be pseudo-replicates, and their deviation (residuals) from the average signal across batches is used to estimate and correct for the batch effects.

Although various methods to correct for batch effects in both the presence and absence of references have been proposed, a systematic comparison of batch correction tools for mass cytometry data is missing. Thus, whether the approach used in this study to align batches on the basis of shared references is the most accurate remains unresolved.

Our pipeline is entirely R-based and does not rely on switching between platforms. Thus, it omits the need for heavy data transfers between online cloud services, graphical user interfaces (GUI), and programming environments for different parts of preprocessing and downstream analysis. As a result, each step in the analysis is fully reproducible and any parameters used throughout can be easily modified and documented. This transition from manual, GUI-based to largely automated, programmatic data processing is indispensable for clinical and other large-scale studies, where sample throughput is high and reproducibility ever so important.

Since its first submission to Bioconductor in 2017, CATALYST has undergone continuous maintenance and development. The most noteworthy changes include implementation of a comprehensive visualization suite based on Nowicka *et al.* ^14^’s workflow for differential discovery; and, the transition from custom data structures to using Bioconductor’s SingleCellExperiment class for differential analysis with Bioconductor v3.11, and for preprocessing with v3.12. Taken together, these developments have transformed CATALYST into a one-stop tool for cytometry data analysis, enabling both data preprocessing and in-depth downstream analysis.

## Methods

### Normalization

***Identification of bead events.*** Commonly, bead events are identified by manual gating on scatter plots of DNA vs. bead channels where DNA show be low, and expression should be high across all bead channels. Instead, we propose a programmatic way to identify beads that includes detection of bead-bead and cell-bead doublets.

**Figure 17.**
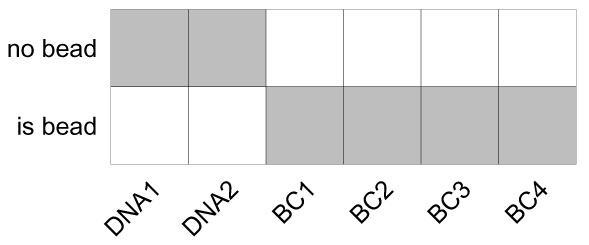
Schematic of the debarcoding scheme used by ‘CATALYST‘’s ‘normCytof()‘ function to identify bead events. Rows correspond to barcodes, columns to DNA and bead channels. Each barcode is either positive (grey) or negative (blank) for a given channel; cells (barcode 11000...) are positive for DNA and negative for bead channels, bead events (barcode 00111...) are negative for DNA and positive for bead channels.

Our normalization strategy leverages the already established SCD algorithm for preliminary tagging of events as beads. In this context, the debarcoding scheme is a 2×(2+ *m*) matrix ( Figure 17). Here, columns correspond to the two DNA channels and *m* barcode channels; rows correspond to barcodes 0 (no bead) and 1 (is bead), where non-bead events are positive for DNA channels only (barcode 11000. . . ), while bead events are negative for DNA and positive for all bead channels (barcode 00111. . . ):

Upon initially assignment of bead events, we apply a *median* ± *n* median absolute deviation (MAD) rule to remove low- and high-signal events from the bead population used for estimating normalization factors. As *n* decreases, bead populations become more narrow and bead-bead doublets are excluded. The extent to which bead populations are trimmed can be adjusted via argument trim (default 5). 

Notably, slight *over*-trimming does not affect normalization. It is therefore recommended to choose a trim value that is small enough to assure removal of doublets at the cost of reduced bead population sizes.

***Correcting for signal-decrease over time.*** To correct for the effect of event acquisition time on signal intensity, we follow the method proposed by Finck *et al.* ^8^. In essence, bead intensities are smoothed using a median sliding-window with width *k* (default 500 bead events). At each timepoint, the slope of a line with intercept zero is computed my minimizing the squared error between smoothed bead and mean bead intensities (= baseline). Alternatively, a reference set of beads from which to compute the baseline can be provided. Slopes for non-bead timepoints are obtained via constant interpolation of these slopes. Here, large slopes correspond to significant deviation from the baseline, and small slopes indicate that the signal is already similar to the baseline. Thus, raw bead counts are normalized by multiplication with the fitted slopes at each timepoint.

### Debarcoding

***Preliminary barcode assignment.*** The debarcoding process commences by assigning each event a preliminary barcode ID. This requires specification of a binary barcoding scheme (or debarcoding key)

$$B=(b_{ij})\in {\left\{0.1\right\}}^{n\times m}$$

where *i* = 1, ..., *n* is the barcode index, *j* = 1, ..., *m* a barcode channel, and *n*, *m* denote the number of unique barcodes and barcoding channels, respectively. Further, let *k _i_* denote the number of positive barcoding channels for barcode *i*: $k_{i}=\sum_{j=1}^{m}b_{ij}.$

If *k _i_* = *k* ∀ *i* = 1, ..., *n* (i.e., every barcode has the same number of positive barcoding channels), the *k* channels with the highest signal in a given event are considered to be positive, the remaining *m* − *k* to be negative. The *separation δ* of positive and negative events is then defined as the difference between the *k*th highest and ( *m* − *k*)th lowest scaled intensity for that event.

***Seperation cutoff estimation.*** When the separation between positive and negative barcoding channels is low, we cannot be confident in the barcode assignment.

**Figure 18.**
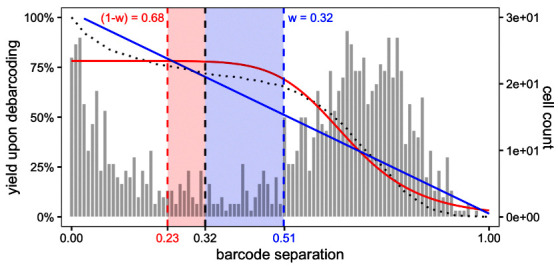
Schematic description of automated separation cutoff estimation. Bar graphs represent the distribution of cells relative to the barcode distance, dotted line scorresponds to yield upon debarcoding as a function of the applied separation cutoff. The yield curve is fitted with a linear regression (blue) and a three parameter log-logistic function (red). The final cutoff estimate (black dashed line) is defined as the mean of estimates derived from both fits, weighted with the goodness of the respective fit.

For the estimation of cutoff parameters, we consider yields upon debarcoding as a function of the applied cutoffs. Commonly, this function will be characterized by an initial weak decline, where doublets are excluded, and subsequent rapid decline in yields to zero. In between, low numbers of counts with intermediate barcode separation give rise to a plateau. To facilitate robust estimation, we fit a linear and a three-parameter log-logistic function ^32^ to the yields function with *drc*’s LL.R function ^33^ ( Figure 18). As an adequate cutoff estimate, we target a point that marks the end of the plateau regime and on-set of yield decline to appropriately balance confidence in barcode assignment and cell yield.

We define the linear model cutoff estimate *c*_LM_ as the value for which the cell yield *Y* has declined to half of the initial Yield *β*_0_:

$$Y=\beta_{0}+c_{linear}\cdot \beta_{1}=\beta_{0}/2\;\Leftrightarrow \;c_{linear}=-\beta_{0}/(2\cdot \beta_{1})$$

where *β*_0_, *β*_1_ are the intercept and slope of the linear model fit, respectively.

We define the log-logistic model cutoff estimate *c*_LLM_ as the value for which the log-logistic function’s decline is minimized relative to its value:

$$c_{log-logistic}=\begin{array}{c} \arg\min\\ x \end{array}\frac{\left|f^{\prime }(x)\right|}{f(x)}>0.1$$

The final cutoff estimate *c* is defined as the weighted mean between these estimates:

$$c=w\cdot c_{linear}+(1-w)\cdot c_{log-logistic}$$

where *w* is the goodness of the linear fit relative to the log-logistic fit:

$$w=\frac{\text{RS}{\text{S}}_{log-logistic}}{\text{RS}{\text{S}}_{log-logistic}+\text{RS}{\text{S}}_{linear}}$$

### Compensation

***Retrieval of real signal.*** As in conventional flow cytometry, we model spillover linearly, with the channel stained for as predictor, and spill-effected channels as response. Thus, the intensity observed in a given channel *j* are a linear combination of its real signal and contributions of other channels that spill into it. Let *I* denote the (unknown) real and *J* the observed signal. Further, let *s _ij_* be the proportion of channel *j* signal that is due to channel *i*, and *w _j_* the set of channels that spill into channel *j*. Then

$$J_{j}=I_{j}+\sum_{i\in w_{j}}s_{ij}$$

In matrix notation, measurement intensities may be viewed as the convolution of real intensities and a spillover matrix $SM=(s_{ij})\in {\mathbb{R}}_{+}^{n\times p},$ where *n* denotes the number of samples (cells) and *p* the number of features (channels): *J* = *I* · *SM*. The real signal *I* can then be retrieved via:

$$I=J\cdot SM^{-1}=J\cdot CM$$

where *SM* ^−1 ^is termed compensation matrix ( *CM*).

While mathematically exact, the solution to this equation will yield negative values, and does not account for the fact that ion counts are strictly non-negative. A computationally efficient way to adress this is to instead use non-negative linear least squares (NNLS), which optimizes the least squares criterion under the constraint of non-negativity:

$$\min\{{\left(J-SM\cdot I\right)}^{T}\cdot (J-SM\cdot I)\}|\;I\geq 0$$

To solve for *I*, we apply the Lawson-Hanson algorithm ^34, 35^ for NNLS implemented in the *nnls* package.

**Figure 19.**
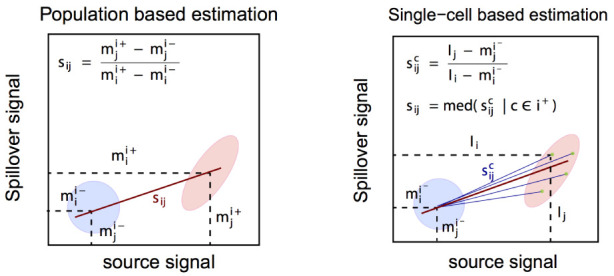
Population versus single-cell based spillover estimation. In a population-based setting (left), spillover is estimated as the slope of a line through the centers of positive (red) and negative (blue) populations. In a single-cell based setting (right), slopes are computed independently for each cell in the positive population, and spillover is estimated as their median.

***Spillover estimation.*** Because any signal not in a single stain experiment’s primary channel *j* results from channel crosstalk, each spill entry *s _ij_* can be approximated by the slope of a linear regression with channel *j* signal as the response, and channel *i* signals as the predictors, where *i* ∈ *w _j_*. computeSpillmat() offers two alternative ways for spillover estimation ( Figure 19).

The default method approximates this slope with the following single-cell derived estimate: Let *i* ^+ ^denote the set of cells that are possitive in channel *i*, and $s_{ij}^{c}$ be the channel *i* to *j* spill computed for a cell *c* that has been assigned to this population. We approximate $s_{ij}^{c}$ as the ratio between the signal in unstained spillover receiving and stained spillover emitting channel, *I _j_* and *I _i_*, respectively. The expected background in these channels, $m_{j}^{-}$ and $m_{i}^{-}$ , is computed as the median signal of events that are i) negative in the channels for which spill is estimated ( *i* and *j*); ii) not assigned to potentionally interacting channels; and, iii) not unassigned, and subtracted from all measurements:

$$s_{ij}^{c}=\frac{I_{j}-m_{j}^{i-}}{I_{i}-m_{i}^{i-}}$$

Each entry *s _ij_* in SM is then computed as the median spillover across all cells *c* ∈ *i* ^+^:

$$s_{ij}=\text{med}(s_{ij}^{c}|c\in i^{+})$$

In a population-based fashion, as done in conventional flow cytometry, *s _ij_* is computed as the slope of a line through the medians (or trimmed means) of stained and unstained populations, $m_{j}^{+}$ and $m_{i}^{+}$ , respectively. Background signal is computed as above and subtracted, according to:

$$s_{ij}=\frac{m_{j}^{+}-m_{j}^{-}}{m_{i}^{+}-m_{i}^{-}}$$

**Figure 20.**
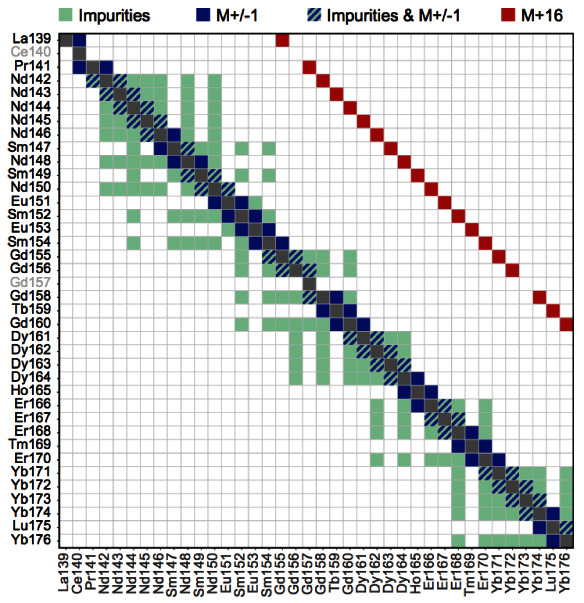
Heatmap of channel interactions expected to exhibit spillover. Included are only interactions that are sensible from a chemical and physical point of view: adjacent mass channels (abundance sensitivity), +16 mass channels (oxidation), and channels measuring isotopes (impurities).

On the basis of their additive nature, spill values are estimated independently for every pair of interacting channels. Hereby, we take into account only interactions that are sensible from a chemical and physical point of view: *M* ± 1 channels (abundance sensitivity), *M* + 16 channels (oxide formation), and channels measuring isotopes (impurities; Figure 20).

Alternatively, interactions = "all" will compute a spill estimate for all *n* · ( *n* − 1) possible interactions, where *n* denotes the number of measurement parameters. Estimates falling below the threshold specified by th will be set to zero. Lastly, note that diagonal entries *s _ii_* = 1 for all *i* ∈ 1, ..., *n*, so that spill is relative to the total signal measured in a given channel.

## Data availability

### Underlying data

The CyTOF data as well as all metadata required to run the full pipeline presented herein are available from Figshare as well as the Tumor Profiler website at https://tu-pro.ch/download/catalyst. 

Figshare: An R-based reproducible and user-friendly preprocessing pipeline for CyTOF data. https://doi.org/10.6084/m9.figshare.c.5063984.v1 ^18^

This project contains the following underlying data:

- CyTOF_acquisition_1-3.fcs (40-Ab panel CyTOF data of 2 blood and 2 tumor samples, and 9 reference samples selected to contain positive and negative populations for each marker included in the study’s Ab-panel. Samples were multiplexed with a 20-well barcoding plate, and obtained from a single run provided as 3 FCS files.)
- normalization_beads.fsc (Beads identified using CATALYST during the normalization step of a previous CyTOF run. – Used as reference beads to correct for changes in signal sensitivity over time across multiple CyTOF runs.)
- ref_bead_counts.csv (A table of mean dual counts for the six different bead channels (columns) obtained from 12 previous acquisitions (rows). – Used as a reference to assess the measurement sensitivity for the current run.)
- debarcoding_scheme.csv (A binary barcoding scheme of 6-choose-3 = 20 barcodes with columns corresponding to barcode channel masses (101, 104, 105, 106, 108, 110) and rows corresponding to barcodes (7 empty, 9 references, 2 PBMC and 2 tumor samples) – Used for single-cell deconvolution of multiplexed of samples.)
- spillover_matrix.csv (A spillover matrix calculated with CATALYST from beads single-stained with each of the 40 antibodies included in the panel used in this study. The matrix contains, for each measurement channel (rows), the percentage of signal received by all other channels (columns). – Used for correction of spillover.)
- ref_cell_counts.csv (A table of the number of cells measured in 7 previous acquisitions, each including 4 cell line, 3 PBMC and 2 tumor references samples (63 samples in total). – Used to asses reference sample cell yields in the current in comparison to previous runs.)
- sample_cell_counts.csv (A table of the number of cells measured in 7 previous acquisitions, each including 2 PBMC and 2 tumor samples each (28 samples in total). – Used to asses sample cell yields in the current in comparison to previous runs.)
- ref_marker_levels.csv (A table of the 98th expression percentiles for each target (columns) across 7 previous acquisitions (rows). – Used to assess the staining efficiency of the current run.)

Data are available under the terms of the Creative Commons Attribution 4.0 International license (CC-BY 4.0). 

## Software availability

Analyses were run in R v4.0.0 ^36^, with Bioconductor v3.11 ^37^, and all software packages used throughout this workflow are publicly available through the Comprehensive R Archive Network ( https://cran.r-project.org) or the Bioconductor project ( http://bioconductor.org). Specific package versions are captured in the following session information:


sessionInfo()



## R version 4.0.0 RC (2020-04-21 r78267)
## Platform: x86_64-apple-darwin17.0 (64-bit)
## Running under: macOS Catalina 10.15.5
##
## Matrix products: default
## BLAS: /Library/Frameworks/R.framework/Versions/4.0/Resources/lib/libRblas.dylib
## LAPACK: /Library/Frameworks/R.framework/Versions/4.0/Resources/lib/libRlapack.dylib
##
## locale:
## [1] en_US.UTF-8/en_US.UTF-8/en_US.UTF-8/C/en_US.UTF-8/en_US.UTF-8
##
## attached base packages:
## [1] parallel  stats4    stats      graphics     grDevices   utils    datasets
## [8] methods base
##
## other attached packages:
##  [1] openCyto_2.0.0              mvtnorm_1.1-1
##  [3] ggcyto_1.16.0               ncdfFlow_2.34.0
##  [5] BH_1.72.0-3                 RcppArmadillo_0.9.900.1.0
##  [7] ggplot2_3.3.2               flowWorkspace_4.0.6
##  [9] flowCore_2.0.1              dplyr_1.0.0
## [11] CATALYST_1.12.2             SingleCellExperiment_1.10.1
## [13] SummarizedExperiment_1.18.2 DelayedArray_0.14.1
## [15] matrixStats_0.56.0          Biobase_2.48.0
## [17] GenomicRanges_1.40.0        GenomeInfoDb_1.24.2
## [19] IRanges_2.22.2              S4Vectors_0.26.1
## [21] BiocGenerics_0.34.0         reshape2_1.4.4
## [23] RColorBrewer_1.1-2          patchwork_1.0.1
## [25] BiocStyle_2.16.0
##
## loaded via a namespace (and not attached):
##   [1] readxl_1.3.1                circlize_0.4.10
##   [3] drc_3.0-1                   plyr_1.8.6
##   [5] igraph_1.2.5                ConsensusClusterPlus_1.52.0
##   [7] splines_4.0.0               BiocParallel_1.22.0
##   [9] fda_5.1.5                   usethis_1.6.1
##  [11] scater_1.16.2               TH.data_1.0-10
##  [13] digest_0.6.25               htmltools_0.5.0
##  [15] viridis_0.5.1               magrittr_1.5
##  [17] CytoML_2.0.5                cluster_2.1.0
##  [19] ks_1.11.7                   openxlsx_4.1.5
##  [21] ComplexHeatmap_2.4.2        RcppParallel_5.0.2
##  [23] R.utils_2.9.2               sandwich_2.5-1
##  [25] cytolib_2.0.3               jpeg_0.1-8.1
##  [27] colorspace_1.4-1            rrcov_1.5-2
##  [29] ggrepel_0.8.2               BiocWorkflowTools_1.14.0
##  [31] haven_2.3.1                 xfun_0.15
##  [33] crayon_1.3.4                RCurl_1.98-1.2
##  [35] jsonlite_1.7.0              hexbin_1.28.1
##  [37] graph_1.66.0                survival_3.2-3
##  [39] zoo_1.8-8                   glue_1.4.1
##  [41] flowClust_3.26.0            gtable_0.3.0
##  [43] nnls_1.4                    zlibbioc_1.34.0
##  [45] XVector_0.28.0              GetoptLong_1.0.2
##  [47] IDPmisc_1.1.20              car_3.0-8
##  [49] BiocSingular_1.4.0          Rgraphviz_2.32.0
##  [51] shape_1.4.4                 DEoptimR_1.0-8
##  [53] abind_1.4-5                 scales_1.1.1
##  [55] Rcpp_1.0.5                  plotrix_3.7-8
##  [57] viridisLite_0.3.0           tmvnsim_1.0-2
##  [59] clue_0.3-57                 mclust_5.4.6
##  [61] foreign_0.8-80              rsvd_1.0.3
##  [63] FlowSOM_1.20.0              tsne_0.1-3
##  [65] httr_1.4.1                  ellipsis_0.3.1
##  [67] R.methodsS3_1.8.0           pkgconfig_2.0.3
##  [69] XML_3.99-0.4                flowViz_1.52.0
##  [71] flowStats_4.0.0             tidyselect_1.1.0
##  [73] rlang_0.4.7                 munsell_0.5.0
##  [75] cellranger_1.1.0            tools_4.0.0
##  [77] generics_0.0.2              ggridges_0.5.2
##  [79] evaluate_0.14               stringr_1.4.0
##  [81] yaml_2.2.1                  knitr_1.29
##  [83] fs_1.4.2                    robustbase_0.93-6
##  [85] zip_2.0.4                   purrr_0.3.4
##  [87] RBGL_1.64.0                 R.oo_1.23.0
##  [89] xml2_1.3.2                  compiler_4.0.0
##  [91] rstudioapi_0.11             beeswarm_0.2.3
##  [93] curl_4.3                    png_0.1-7
##  [95] tibble_3.0.3                pcaPP_1.9-73
##  [97] stringi_1.4.6               forcats_0.5.0
##  [99] lattice_0.20-41             Matrix_1.2-18
## [101] vctrs_0.3.2                 pillar_1.4.6
## [103] lifecycle_0.2.0             BiocManager_1.30.10
## [105] GlobalOptions_0.1.2         BiocNeighbors_1.6.0
## [107] corpcor_1.6.9               data.table_1.12.8
## [109] cowplot_1.0.0               bitops_1.0-6
## [111] irlba_2.3.3                 R6_2.4.1
## [113] latticeExtra_0.6-29         bookdown_0.20
## [115] KernSmooth_2.23-17          gridExtra_2.3
## [117] RProtoBufLib_2.0.0          rio_0.5.16
## [119] vipor_0.4.5                 codetools_0.2-16
## [121] MASS_7.3-51.6               gtools_3.8.2
## [123] rjson_0.2.20                withr_2.2.0
## [125] mnormt_2.0.1                multcomp_1.4-13
## [127] GenomeInfoDbData_1.2.3      hms_0.5.3
## [129] grid_4.0.0                  rmarkdown_2.3
## [131] DelayedMatrixStats_1.10.1   carData_3.0-4
## [133] Rtsne_0.15    	             git2r_0.27.1
## [135] base64enc_0.1-3             ellipse_0.4.2
## [137] ggbeeswarm_0.6.0


## Consent

Written informed consent for publication of the tumor and blood samples was obtained from the patients (BASEC-Nr.2018-02050, approved by the Kantonal Ethics Commisions of Zurich and Basel).

## Author Information

HCL and SC contributed equally to the scientific content of this article and share first authorship. MDR and BB act as co-senior authors.

## Tumor Profiler Consortium members and affiliations

Rudolf Aebersold ^2^, Faisal S Al-Quaddoomi ^7, 14^, Jonas Albinus ^6^, Ilaria Alborelli ^22^, Sonali Andani ^2, 5, 14, 24, 27^, Per-Olof Attinger ^9^, Marina Bacac ^13^, Daniel Baumhoer ^22^, Beatrice Beck-Schimmer ^34^, Niko Beerenwinkel ^3^, Christian Beisel ^3^, Lara Bernasconi ^25^, Anne Bertolini ^7, 14^, Bernd Bodenmiller ^31^, Ximena Bonilla ^2, 5, 14, 24^, Ruben Casanova ^31^, Stéphane Chevrier ^31^, Natalia Chicherova ^7, 14^, Maya D’Costa ^8^, Esther Danenberg ^32^, Natalie Davidson ^2, 5, 14, 24^, Monica-Andreea Drăgan ^3^, Reinhard Dummer ^26^, Stefanie Engler ^31^, Martin Erkens ^11^, Katja Eschbach ^3^, Cinzia Esposito ^32^, André Fedier ^15^, Pedro Ferreira ^3^, Joanna Ficek ^2, 5, 14, 24^, Anja L Frei ^27^, Bruno Frey ^10^, Sandra Goetze ^6^, Linda Grob ^7, 14^, Gabriele Gut ^32^, Detlef Günther ^4^, Martina Haberecker ^27^, Pirmin Haeuptle ^1^, Viola Heinzelmann-Schwarz ^15, 21^, Sylvia Herter ^13^, Rene Holtackers ^32^, Tamara Huesser ^13^, Anja Irmisch ^26^, Francis Jacob ^15^, Andrea Jacobs ^31^, Tim M Jaeger ^9^, Katharina Jahn ^3^, Alva R James ^2, 5, 14, 24^, Philip M Jermann ^22^, André Kahles ^2, 5, 14, 24^, Abdullah Kahraman ^14, 27^, Viktor H Koelzer ^27^, Werner Kuebler ^23^, Jack Kuipers ^3^, Christian P Kunze ^20^, Christian Kurzeder ^18^, Kjong-Van Lehmann ^2, 5, 14, 24^, Mitchell Levesque ^26^, Sebastian Lugert ^8^, Gerd Maass ^10^, Philipp Markolin ^2, 5, 14, 24^, Julien Mena ^2^, Ulrike Menzel ^3^, Nicola Miglino ^1^, Emanuela S Milani ^6^, Holger Moch ^27^, Simone Muenst ^22^, Riccardo Murri ^33^, Charlotte KY Ng ^22, 30^, Stefan Nicolet ^22^, Marta Nowak ^27^, Patrick GA Pedrioli ^2^, Lucas Pelkmans ^32^, Salvatore Piscuoglio ^15, 22^, Michael Prummer ^7, 14^, Mathilde Ritter ^15^, Christian Rommel ^11^, María L Rosano-González ^7, 14^, Gunnar Rätsch ^2, 5, 14, 24^, Natascha Santacroce ^3^, Jacobo Sarabia del Castillo ^32^, Ramona Schlenker ^12^, Petra C Schwalie ^11^, Severin Schwan ^9^, Tobias Schär ^3^, Gabriela Senti ^25^, Franziska Singer ^7, 14^, Sujana Sivapatham ^31^, Berend Snijder ^2^, Bettina Sobottka ^27^, Vipin T Sreedharan ^7, 14^, Stefan Stark ^2, 5, 14, 24^, Daniel J Stekhoven ^7, 14^, Tinu M Thomas ^2, 5, 14, 24^, Markus Tolnay ^22^, Vinko Tosevski ^13^, Nora C Toussaint ^7, 14^, Mustafa A Tuncel ^3^, Audrey Van Drogen ^6^, Marcus Vetter ^17^, Tatjana Vlajnic ^22^, Sandra Weber ^25^, Walter P Weber ^16^, Rebekka Wegmann ^2^, Michael Weller ^29^, Fabian Wendt ^6^, Norbert Wey ^27^, Andreas Wicki ^1, 15, 19^, Bernd Wollscheid ^6^, Shuqing Yu ^7, 14^, Johanna Ziegler ^26^, Marc Zimmermann ^2, 5, 14, 24^, Martin Zoche ^27^, Gregor Zuend ^28^
