# Supplementary material for: An R-based reproducible and user-friendly preprocessing pipeline for CyTOF data
Source: F1000Res. 2022 Aug 8;9:1263. Originally published 2020 Oct 22. [Version 2] doi: 10.12688/f1000research.26073.2 (PMC9411975; doi:10.12688/f1000research.26073.2)
Supplement: Peer review history

# Peer review: Reviewer response for version 2

**DOI:** 10.5256/f1000research.135465.r146797

**Published:** 2022-08-25

**Authors:** Marie Trussart

**Affiliations:** 1 Bioinformatics Division, Walter and Eliza Hall Institute of Medical Research, Parkville, Vic, Australia

Thanks for the updated version and for assessing the suggestions and issues mentioned in the first report. 

 I think this revised version is easier to follow and clearer with the updates provided.

 One last comment would be on the batch alignment section, the Figure 17 selected is not showing the 7 different markers distribution to compare the expression distributions before and after batch correction and I think it would be useful in the assessment of this correction.

Is the rationale for developing the new method (or application) clearly explained?

Yes

Is the description of the method technically sound?

Yes

Are the conclusions about the method and its performance adequately supported by the findings presented in the article?

Yes

If any results are presented, are all the source data underlying the results available to ensure full reproducibility?

Yes

Are sufficient details provided to allow replication of the method development and its use by others?

Partly

Reviewer Expertise:

I work in Bioinformatics and especially in the normalization and batch correction of CyTOF datasets.

I confirm that I have read this submission and believe that I have an appropriate level of expertise to confirm that it is of an acceptable scientific standard.

# Peer review: Reviewer response for version 1

**DOI:** 10.5256/f1000research.28774.r73588

**Published:** 2020-12-01

**Authors:** Sean C. Bendall, Felix Hartmann

**Affiliations:** 1 School of Medicine, Stanford University, Palo Alto, CA, USA; 2 Immunology Graduate Program, Stanford University, Stanford, CA, USA

In their manuscript, Crowell & Chevrier et al. present a novel workflow to preprocess mass cytometry (CyTOF) datasets in R. The presented pipeline is a useful update on earlier publications and packages, including the authors' CATALYST package which is clearly stated. Overall, I find this to be a valuable tool that brings together different functionalities into a unified workflow that enables reproducible and comprehensive preprocessing of this data type. The different steps and approaches are well described and illustrated. Especially, the inclusion of a functionality to perform live cell gating without having to switch platforms is much appreciated, although its current implementation could be improved:

1. Gating on cells: Cells are first identified using an elliptical gate to exclude the two lowest density percentiles. Firstly, this plot relies on two DNA channels (whose information is likely redundant) and wasn’t directly applicable to alternative DNA stains (e.g. rhodium). Furthermore, I am wondering whether this approach might exclude for example a fraction of cycling cells or preferentially exclude cell types or states with increased chromatin accessibility and therefore higher DNA signal?
2. Gating on live cells: The approach suggested by the authors worked well on my test data, however, it takes a while to manually adjust values for every file to fit the gates closely to the data. While I see the value of automating this step, I also think that some manual gating could simplify the process and further increase downstream data quality. Potentially, the authors could adopt an approach like the gate_draw function from the CytoRSuite library.  
3. Compensation: The workflow includes compensation as a preprocessing step which the authors have shown in separate publications to improve data quality, but which is currently not routinely performed by many researchers working using mass cytometry. I, therefore, assume that most users of this pipeline would be relying on published spillover matrices that reflect estimates of isotope purity and oxidation. While I agree with the usefulness of this function, I believe that adding additional quality control functions could improve acceptance of and trust in this approach. For example, in flow cytometry, overcompensation is often easily spotted by the occurrence of overly negative values, however, using their NNLS approach this is not readily apparent in compensated mass cytometry data. It would be very helpful to have a quality metric that would alert users to such potential issues introduced by the compensation step.

In addition, testing this pipeline on some in-house generated data, a few minor issues occurred which should be addressed:

1. While this might only be needed in rare cases, a function to rename channels and potentially match these names across multiple fcs files could enhance the adaptability of this package. In my test case, conflicting channel names prevented the import of the files into the workflow. In other cases, it might help to match channel names between batches. The authors could look to the premessa package for inspiration.
2. The authors have incorporated various options for DNA channels which is much appreciated. My test data had been stained with a rhodium intercalator. Specifying this worked well, only the res$scatter function seems to ignore this choice and instead seems to default to iridium DNA intercalators.
3. Sample specific debarcoding is appreciated. Figure 6 and the plotYields function return a debarcoding percentage. I believe this percentage refers to percent of initial assignments, but it is not specifically stated. It might be helpful to get a feeling of the percentage of cells (out of total cells) that are assigned after refining the initial assignments.

Is the rationale for developing the new method (or application) clearly explained?

Yes

Is the description of the method technically sound?

Yes

Are the conclusions about the method and its performance adequately supported by the findings presented in the article?

Yes

If any results are presented, are all the source data underlying the results available to ensure full reproducibility?

Yes

Are sufficient details provided to allow replication of the method development and its use by others?

Yes

Reviewer Expertise:

Single cell proteomics, Mass Cytometry, Immunology, Stem Cell biology

We confirm that we have read this submission and believe that we have an appropriate level of expertise to confirm that it is of an acceptable scientific standard.

# Response

**Published:** 2022-06-23

**Authors:** Helena Crowell

**Affiliations:** University of Zurich, Switzerland

1. *Gating on cells: Cells are first identified using an elliptical gate to exclude the two lowest density percentiles. Firstly, this plot relies on two DNA channels (whose information is likely redundant) and wasn’t directly applicable to alternative DNA stains (e.g. rhodium). Furthermore, I am wondering whether this approach might exclude for example a fraction of cycling cells or preferentially exclude cell types or states with increased chromatin accessibility and therefore higher DNA signal?*
  The first gating step is indeed performed on two DNA channels which contains redundant information. However, this approach is commonly used in the mass cytometry field to exclude debris and cell doublets. By modifying the quantile and the target value defining the center of the ellipse, the user can control how many cells are excluded from the gate and ensure that most cycling cells are kept in the analysis.
  To gate on alternative DNA stains, a different pair of channels could be assigned to the “dna” variable in the corresponding code chunk. In the case of a single DNA channel, a one-dimensional gating (i.e., thresholding) could be applied (as opposed to the currently used elliptical gate). We have added a comment mentioning this to the text under “Gating on cells”.
2. *Gating on live cells: The approach suggested by the authors worked well on my test data, however, it takes a while to manually adjust values for every file to fit the gates closely to the data. While I see the value of automating this step, I also think that some manual gating could simplify the process and further increase downstream data quality. Potentially, the authors could adopt an approach like the gate_draw function from the CytoRSuite library.*
  Indeed, the approach depicted in this paper works well in cases where a limited number of samples are included in a run and when the live/dead cell profile is well defined and consistent between samples. The process can indeed be tedious when hundreds of samples are included in a run or when the live/dead cell profile is more complex. In the latter case, including a function similar to gate_draw function from CytoRSuite could be helpful. However, we here aimed at proposing an automated pipeline; manual gating would defeat this purpose.
  As a side note: We have attempted applying CytoRSuite, however, encountered several confusing issues that we’ve been unable to resolve: The CytoRSuite site ( https://dillonhammill.github.io/CytoRSuite) lists a GH repository that no-longer exists; we could find an installable version at https://github.com/gfinak/cytoRSuite (is this the same?) but ‘drawGate()’ gave an error that we have not been able to resolve; meanwhile, any of CytoRSuite, cytoRSuite and cytoSuite (from which the latter has been forked) have not been changed in 4 years. Taken together, this gave us the feeling that the tool is no longer maintained and likely to be inapplicable with current versions of R and Bioconductor.
  Of course, there may be other tools available at this point for manual gating, and we leave it to the user to incorporate these into their workflow should that be of interest. A possible strategy then might be to i) perform manual gating and export the resulting gates into a table (gating scheme); ii) apply that scheme in an automated fashion (e.g., using the code we presented); and, iii) do manual adjustments to refine gates according to the current experiment.
3. *Compensation: The workflow includes compensation as a preprocessing step which the authors have shown in separate publications to improve data quality, but which is currently not routinely performed by many researchers working using mass cytometry. I, therefore, assume that most users of this pipeline would be relying on published spillover matrices that reflect estimates of isotope purity and oxidation. While I agree with the usefulness of this function, I believe that adding additional quality control functions could improve acceptance of and trust in this approach. For example, in flow cytometry, overcompensation is often easily spotted by the occurrence of overly negative values, however, using their NNLS approach this is not readily apparent in compensated mass cytometry data. It would be very helpful to have a quality metric that would alert users to such potential issues introduced by the compensation step.*
  These are all very good points and legitimate concerns. As indicated in the original paper, the spillover matrix used to compensate mass cytometry data should be calculated based on the antibodies included in the panel. We should stress here that, based on the single-stained bead acquisition approach presented in the original paper, the experimental procedure required to generate a compensation matrix is fairly straightforward and can be achieved rapidly. Using a previously published spillover matrix is a risky strategy, which can indeed lead to inaccurate compensation. The user should instead first run the compensation in classic mode and perform a visual inspection to ensure no overcompensation can be detected before using the NNLS method. This is a valuable option to avoid this specific type of artefact. Automating this step is a good suggestion, but is out of the scope of this publication and comes with some disadvantages. The risk we see is that this process could be imperfect and potentially misleading for the user. Indeed, such an approach would only identify overcompensation in channels where a single positive population is present but not in the case of a double positive population. In other words, it will highly depend on the user’s data type. Furthermore, it would not identify under-compensated signals. As a consequence, providing an approach to alert users of potential issues would likely be imperfect and could give a wrong impression that the data are correctly compensated if no alert is raised, which is not necessarily the case. Moreover, to the best of our knowledge, such an approach also doesn’t exist in fluorescent flow cytometry, most likely due to the fact that ensuring accurate compensation on a fully stained data set is a challenging task. We should also mention that the spillover coefficients in mass cytometry rarely exceed 4% and therefore the consequences of a slight over or under-compensation are less important in mass cytometry than in flow cytometry.

Minor comments:

1. *While this might only be needed in rare cases, a function to rename channels and potentially match these names across multiple fcs files could enhance the adaptability of this package. In my test case, conflicting channel names prevented the import of the files into the workflow. In other cases, it might help to match channel names between batches. The authors could look to the premessa package for inspiration.*
  We very much appreciate this comment as we have encountered various discrepancies between panels, especially in long-term projects. To date, we have used a custom R script to i) read in files separately; ii) fix panels according to a reference file (i.e., removing/adding additional/missing channels); and, iii) write out a new set of FCS files with concordant panels.
  However, this solution is suboptimal as it leads to a duplication of files (or, in case the original files were overwritten, the process would no longer be reproducible). Similarly, a GUI solution (as ‘premessa’) would defeat the purpose of providing an automated, reproducible preprocessing solution. Thus, taken together, we propose (and have now implemented) the following strategy:
  > ‘prepData’ now exposes additional arguments to be passed to ‘flowCore::read.FCS’ via ‘...’
  > ‘read.FCS’ provides an argument ‘channel_alias’: “an optional ‘data.frame’ used to provide the alias of the channels to standardize and solve the discrepancy across FCS files. [...]”
  > independent of whether or not this option is used, ‘prepData’ will check whether panels (FCS channel names) match between files:
  - in case of any discrepancy, the newly added ‘fix_chs’ argument
    will be used to determine how to resolve discrepancies
  - “all” will keep all channels (i.e., the union across files);
    any missing channels will be added to the respective samples,
    and a channels x samples matrix is stored in the object to track
    which channels were present in which samples originally
  - “common” will keep shared channels (i.e., the intersection across files); any other channels will be dropped from the respective files
  - ‘prepData’ will, in any case, return a ‘SingleCellExperiment’, i.e.,
    no altered FCS files or ‘flowFrame’s will be written out / returned
2. *The authors have incorporated various options for DNA channels which is much appreciated. My test data had been stained with a rhodium intercalator. Specifying this worked well, only the res$scatter function seems to ignore this choice and instead seems to default to iridium DNA intercalators.*
  We thank the reviewer for noticing this. Indeed, while the workflow allows for specification of the DNA channels used (via variable ‘dna’), these were fixed internally in CATALYST’s ‘normCytof()’ function. We have added an additional argument to allow passing custom DNA channel masses (default ‘dna = c(191, 193)’ for Ir191/3; for Rh103, the argument would be ‘dna = 103’ instead); the output scatter plot of DNA vs. bead intensities (‘res$scatter’) is now generated based on the first matching DNA channel (see updated ‘?normCytof’ documentation).
3. *Sample specific debarcoding is appreciated. Figure 6 and the plotYields function return a debarcoding percentage. I believe this percentage refers to percent of initial assignments, but it is not specifically stated. It might be helpful to get a feeling of the percentage of cells (out of total cells) that are assigned after refining the initial assignments.*
  That is correct: As in the original Finck et al. outputs (a MATLAB application), yields (left-hand y-axis) correspond to the proportion of cells that would be retained upon applying a given cutoff (x-axis). In Figure 8, we compare the absolute barcode population sizes before vs. after debarcoding. Analogously, it would be straightforward for users to generate such a barplot from cell counts obtained when applying various thresholding schemes (e.g., no filtering compared to global vs. sample-specific separation cutoffs).

# Peer review: Reviewer response for version 1

**DOI:** 10.5256/f1000research.28774.r73591

**Published:** 2020-11-05

**Authors:** Marie Trussart

**Affiliations:** 1 Bioinformatics Division, Walter and Eliza Hall Institute of Medical Research, Parkville, Vic, Australia

The manuscript is presenting an updated version of the CATALYST package for preprocessing Cytof data. It is well detailed with several examples and has been updated based on the Bioconductor SingleCellExperiment class. Every step of preprocessing is clearly stated and illustrated to guide the user on the different steps to process their data. Also, new quality checks are being reviewed to explore the quality of the data.

 I provided below some feedback to make the manuscript clearer and some suggestions to address some issues I encountered:

1. It will be useful to define clearly what are the differences between successive acquisitions, single CyTOF run and batch.
2. The different samples and runs listed through the different examples could be better presented with a table containing all runs and samples. In the data description, it explains that "The dataset used in this study was obtained from a single CyTOF run containing nine references, three blood samples and three tumor samples barcoded with a 20-well barcoding plate". However in the quality checks section; additional data is being analyzed which makes it confusing, coming from additional runs, sometimes from 7 runs or other times from 8 runs.
3. Batch alignment:
  Could you provide an additional plot showing the effect of applying this correction factor?
  How are you assessing the performance of your batch alignment method?
4. argument norm_to in the normCytof() function: give explanations on how it being computed when giving reference beads, especially how does it compute the new baseline, does it takes into account both the beads from the reference and current by averaging both? 
  Can it be used to normalize data from different batches? If so how does it deal with distinguishing times and ordering the beads and time which would be similar in separated batches?
5. Figure 4: Could you please give more explanations on how to assess run sensitivity and how does the user decide what is acceptable and what is not?
  Also, you need to load the library(reshape2) to run this part.
6. The wrap_plots function is missing here.
7. I got an error when running the QC on reference cell counts.  "Error: Can't combine `1$CellLine_R1` and `2$CellLine_R1` ."

**Minor comments:**

1. When running the code using the data provided, the directory name should be modified to "CyTOF_acquisition_1-3.FCS/" instead of data
  fcs <- list.files("CyTOF_acquisition_1-3.FCS/", "acquisition", full.names = TRUE)
  Also, it should be specified that the directory name containing all the data should be called "data" and it refers to the directory name, or an alternative is to have the local directory "." instead of data like in here:
  # specify path to reference beads
  ref_beads <- file.path(".", "normalization_beads.fcs")
2. Introduction:
  "an important step is to correct for batch effects, which can be achieved by including a shared control sample in each independent batch ^11, 12 ^" Add CytofRUV reference mentioned in the discussion.
3. "In our example, barcode identifiers include each sample’s type (CellLine, PBMC or Tumor), group (R for reference or S for sample of interest), and replicate number; and follow a consistent naming scheme: We can easily extract these components and store them in the SCE’s cell metadata (colData)". The example selected is not the best one, as it not showing any differences between the 6 first row.
4. There is a typo in the legend of some figures like figure 17: "previously" acquired runs.

Is the rationale for developing the new method (or application) clearly explained?

Yes

Is the description of the method technically sound?

Yes

Are the conclusions about the method and its performance adequately supported by the findings presented in the article?

Yes

If any results are presented, are all the source data underlying the results available to ensure full reproducibility?

Yes

Are sufficient details provided to allow replication of the method development and its use by others?

Partly

Reviewer Expertise:

I work in Bioinformatics and especially in the normalization and batch correction of CyTOF datasets.

I confirm that I have read this submission and believe that I have an appropriate level of expertise to confirm that it is of an acceptable scientific standard, however I have significant reservations, as outlined above.

# Response

**Published:** 2022-06-23

**Authors:** Helena Crowell

**Affiliations:** University of Zurich, Switzerland

1. *It will be useful to define clearly what are the differences between successive acquisitions, single CyTOF run and batch.*
  Indeed, the meaning behind the concepts of successive acquisitions, single CyTOF run and batch was not fully clear and these terms were not used in a consistent way. A “CyTOF run” corresponds to an independent experiment where samples are stained and acquired simultaneously on the CyTOF. We replaced the term run with experiment to clarify the meaning. Each CyTOF experiment corresponds to one “batch” and this term is used to refer to the batch correction which is performed on the different CyTOF experiments. The data from a single CyTOF experiment are usually acquired over multiple “successive acquisitions”, each leading to the generation of a single FCS file. We also made the use of these terms consistent throughout the paper.
2. *The different samples and runs listed through the different examples could be better presented with a table containing all runs and samples. In the data description, it explains that "The dataset used in this study was obtained from a single CyTOF run containing nine references, three blood samples and three tumor samples barcoded with a 20-well barcoding plate". However in the quality checks section; additional data is being analyzed which makes it confusing, coming from additional runs, sometimes from 7 runs or other times from 8 runs.*
  The pipeline described in this paper was designed to preprocess CyTOF data acquired over a long period of time with a focus on ensuring data consistency over time. The aim of the workflow is to guide the readers through the preprocessing steps required to convert FCS files obtained in a given CyTOF experiment to a format suitable for downstream analysis, while presenting key quality checks to ensure the reliability of the data generated in the experiment of interest. Therefore, the whole analysis is based on a dataset obtained from a single CyTOF experiment, which is benchmarked against data acquired during a preparatory phase. For consistency reasons, we included now systematically the data from seven previous CyTOF experiments to benchmark the data of the CyTOF experiment preprocessed in this paper.
3. *Batch alignment: Could you provide an additional plot showing the effect of applying this correction factor? How are you assessing the performance of your batch alignment method?*
  The batch alignment presented in this paper is based on a linear scaling based on a percentile, using references as anchoring points, similar to a previously published method (Schuyler et al, 2019). To assess the performance of our batch alignment method, we have now included a figure to compare the expression distributions before and after batch correction (including their 98th percentiles and those of the references). As intended, 98th percentiles align with the references’ upon correction, while expression distributions remain virtually unchanged.
4. *argument norm_to in the normCytof() function: give explanations on how it being computed when giving reference beads, especially how does it compute the new baseline, does it take into account both the beads from the reference and current by averaging both?*
  Normalization using reference beads follows the methodology originally introduced in Finck et al. (2013): The baseline is computed as the mean intensity of the reference beads only, not including the current experiment. Would the average be taken over both, intensities would not be aligned between current and reference experiment. While the statement “[...] We provide the path to a set of reference beads (argument `norm_to`) that are used to compute baseline intensities for normalization” explains this only briefly, we believe that the method is well established and readers should refer to the original publication for more detail.
*Can it be used to normalize data from different batches? If so how does it deal with distinguishing times and ordering the beads and time which would be similar in separate batches?*
  Yes, certainly. The normalization aims at correcting the signal time-drift due to progressive loss of sensitivity during acquisition. This is a technical effect that is independent of batch effects, and should be accounted for regardless of whether or not batch effects are present: these should be corrected for downstream analysis.
  Events from different FCS files (independent of whether these are different acquisitions of the same experiment or batches) are concatenated. How event times are dealt with depends on prepData()’s input arguments. When by_time = TRUE, files are ordered according to their acquisition time (stored under each flowFrame’s $BTIM description field). Otherwise, they are kept in the order provided by the input metadata table (argument md).
5. *Figure 4: Could you please give more explanations on how to assess run sensitivity and how does the user decide what is acceptable and what is not?*
  Instrument sensitivity is an important parameter that should be closely monitored. This parameter is assessed during the tuning but those data cannot be easily exported and compared between experiments. The aim was to take advantage of the beads, which are run together with the samples to report on instrument sensitivity. Figure 3 provides key information regarding how the sensitivity evolves during the run, while the point of Figure 4 is to show how the average sensitivity evolves from one experiment to another. Instrument sensitivity varies from machine to machine and deciding what is acceptable will depend on the requirements of the users. The point of this plot was to offer an option for the user to easily identify in case the sensitivity is getting low compared to previous experiments, and to make a link between the quality of the data generated in a specific experiment with the sensitivity of the instrument.
*Also, you need to load the library(reshape2) to run this part.*
  Yes, thank you for catching this; we’ve added reshape2 to the list of dependencies, and it is now loaded along the other required libraries.
6. *The wrap_plots function is missing here.*
  Yes, thank you for catching this; we’ve added patchwork to the list of dependencies, and it is now loaded along the other required libraries.
7. *I got an error when running the QC on reference cell counts. "Error: Can't combine `1$CellLine_R1` and `2$CellLine_R1`."*
  True, thank you; I could reproduce this with the current R and package versions. It has been fixed by converting the ‘run’ object of class ‘table’ to call ‘integer’ using c().

Minor comments:

1. *When running the code using the data provided, the directory name should be modified to "CyTOF_acquisition_1-3.FCS/" instead of data:*
* fcs <- list.files("CyTOF_acquisition_1-3.FCS/", "acquisition", full.names = TRUE)*
  We are not sure we understand this comment. ‘list.files(path, pattern, …)’ expects the first argument to be a directory (where the FCS files are located), not the file names themselves (“xxx.FCS”).
*Also, it should be specified that the directory name containing all the data should be called "data" and it refers to the directory name, or an alternative is to have the local directory "." instead of data like in here:*
*# specify path to reference beads*
* ref_beads <- file.path(".", "normalization_beads.fcs")*
  Thank you, yes, we forgot to mention that in the presented code all data used throughout the workflow is expected to sit inside a “data” subdirectory relative to where the .Rmd file is being run. We have now added a note explaining this in the 2nd paragraph under “Results”.
2. *Introduction: "an important step is to correct for batch effects, which can be achieved by including a shared control sample in each independent batch" Add CytofRUV reference mentioned in the discussion.*
  We updated the reference to CytofRUV to the new version of the manuscript published in eLife and added it to the introduction.
3. *"In our example, barcode identifiers include each sample’s type (CellLine, PBMC or Tumor), group (R for reference or S for sample of interest), and replicate number; and follow a consistent naming scheme: We can easily extract these components and store them in the SCE’s cell metadata (colData)". The example selected is not the best one, as it not showing any differences between the 6 first row.*
  True. We have modified the example to sample 10 unique ‘sample’ entries (= type_group) for which to display the ‘colData’.
4. *There is a typo in the legend of some figures like figure 17: "previously" acquired runs.*
  Thanks for pointing out this typo, which was corrected in the corresponding figures.
